# Supplementary material for: Comprehensive Analysis of the Immune Response to SARS-CoV-2 Epitopes: Unveiling Potential Targets for Vaccine Development
Source: Biology (Basel). 2025 Jan 14;14(1):67. doi: 10.3390/biology14010067 (PMC11761943; doi:10.3390/biology14010067)
Supplement: Supplementary file 1 [file biology-14-00067-s001.zip › biology-3330086- supplementary material.pdf]

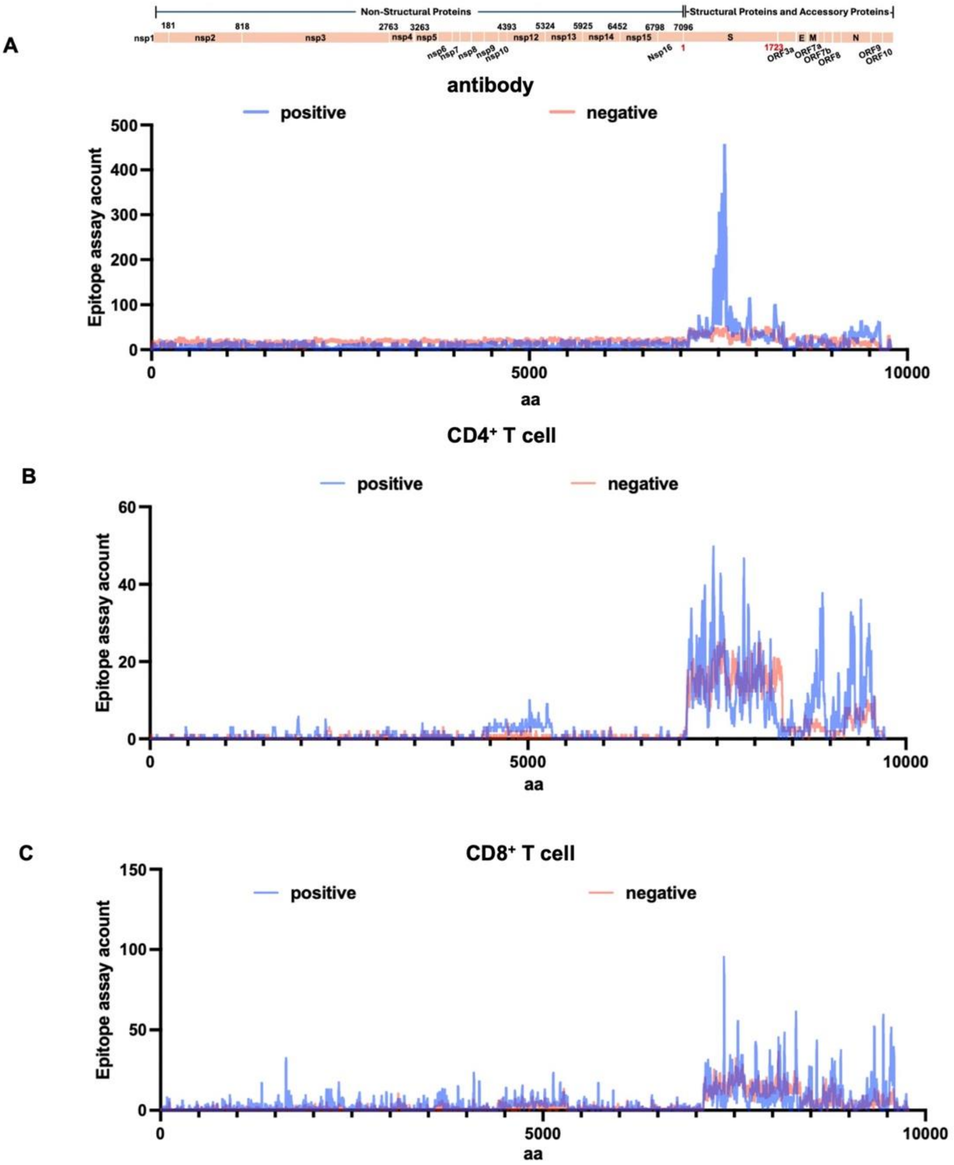

Figure S1. The number of SARS-CoV-2 epitopes assay account records. A. The number of antibody epitopes assay account. **B.** The number of CD4+ T cell epitopes assay account. **C.** The number of CD8+ T cell epitopes assay account.

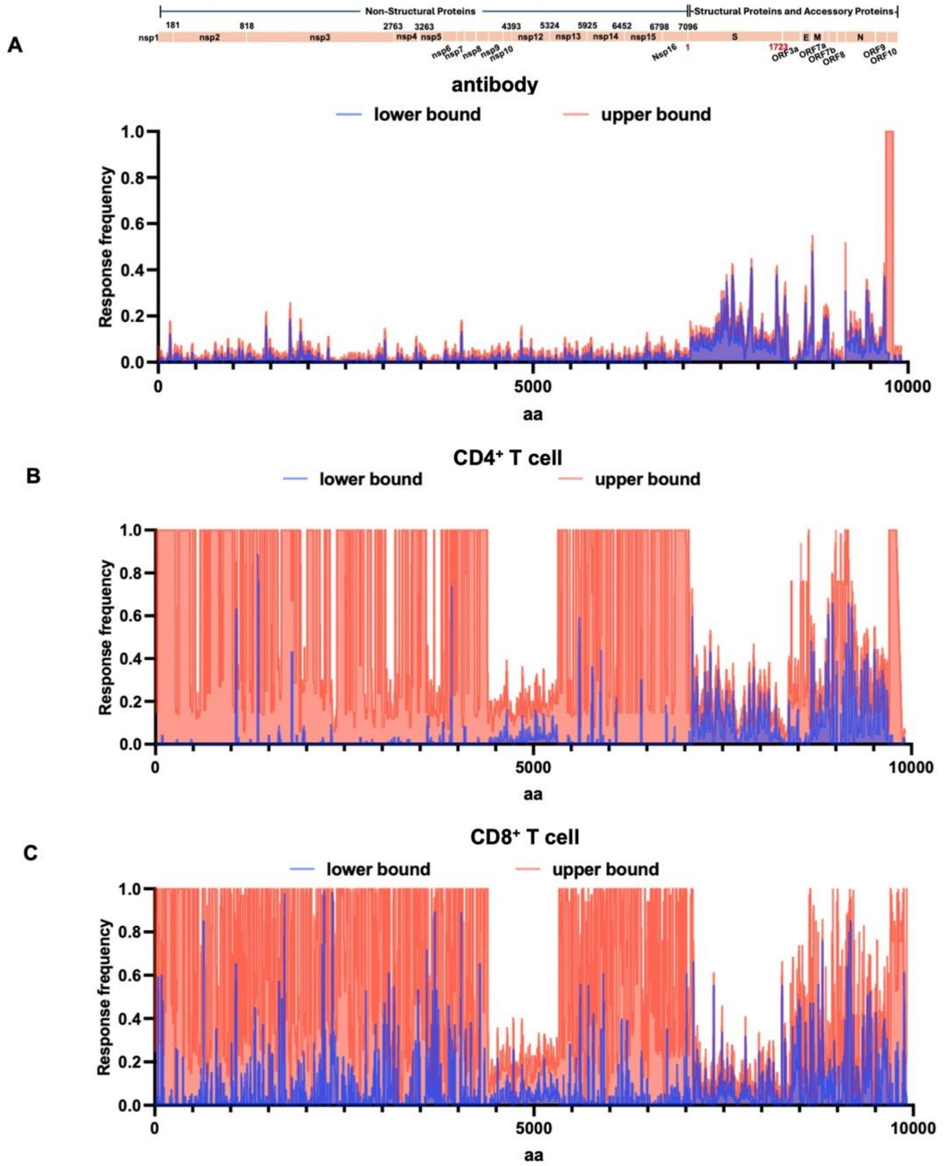

Figure S2. The lower and upper bounds of the 95% confidence interval (CI) for the response frequency (RF) at each target protein position. A. The lower and upper bounds of the 95% confidence interval (CI) for the antibody response frequency (RF) at each target protein of SARS-CoV-2. **B.** The lower and upper bounds of the 95% confidence interval (CI) for the CD4$^+$ T cell response frequency (RF) at each target protein of SARS-CoV-2. **C.** The lower and upper bounds of the 95% confidence interval (CI) for the CD8$^+$ T cell response frequency (RF) at each target protein of SARS-CoV-2.

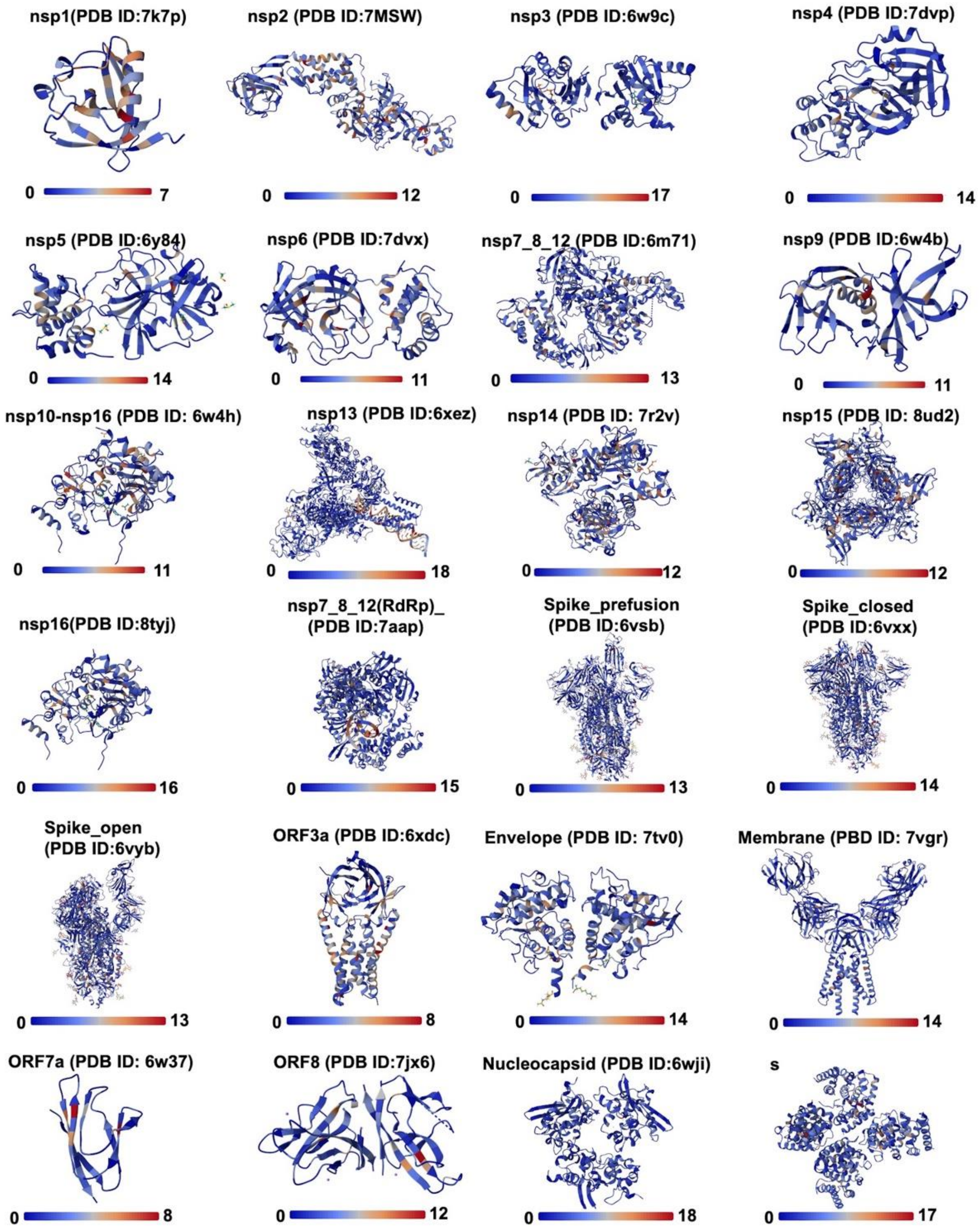

Figure S3. Structure-based network analysis of the SARS-CoV-2 proteome.

Table S1. List of sequences of 33 representative alpha- and beta-coronavirus genome and protein sequences used in this analysis.

accession numbers	isolate	genus	sub-genus
NC_005831	CoV-NL63	Alpha coronavirus	Setracovirus
KY983587	CoV-229E	Alpha coronavirus	Duvinacovirus
MW246798.1	EriCoV	Beta coronavirus	Merbecovirus
NC_019843	MERS-CoV	Beta coronavirus	Merbecovirus
MH002342.1	Pi-BatCoV-HKU5	Beta coronavirus	Merbecovirus
EF065505.1	Ty-BatCoV-HKU4	Beta coronavirus	Merbecovirus
AY278741.1	SARS-CoV	Beta coronavirus	Sarbecovirus
MN908947.3	SARS-CoV-2	Beta coronavirus	Sarbecovirus
MN996532.2	bat_RATG13	Beta coronavirus	Sarbecovirus
MG772934.1	ZXC21	Beta coronavirus	Sarbecovirus
MT072864.1	GX-P2V	Beta coronavirus	Sarbecovirus
MT040336.1	GX-P5E	Beta coronavirus	Sarbecovirus
MT040335.1	GX-P5L	Beta coronavirus	Sarbecovirus
MT040334.1	GX-P1E	Beta coronavirus	Sarbecovirus
MT040333.1	GX-P4L	Beta coronavirus	Sarbecovirus
MT072865.1	GX-P3B	Beta coronavirus	Sarbecovirus
MT121216.1	MP789	Beta coronavirus	Sarbecovirus
KT444582.1	bat-WIV16	Beta coronavirus	Sarbecovirus
KF367457.1	WIV1	Beta coronavirus	Sarbecovirus
KP886808.1	YNLF_31C	Beta coronavirus	Sarbecovirus
FJ588686.1	Rs672	Beta coronavirus	Sarbecovirus
FJ211859.1	recombinant strain	Beta coronavirus	Sarbecovirus
AY572034.1	Civet007	Beta coronavirus	Sarbecovirus
AY686863.1	CivetA022	Beta coronavirus	Sarbecovirus
AY686864.1	CivetB039	Beta coronavirus	Sarbecovirus
KF636752.1	Bat Hp-BetaCoV	Beta coronavirus	Hibecovirus
MT350598.1	Ro-BaCoV GCCDC1	Beta coronavirus	Nobecovirus
MG762674.1	Ro-BatCoV HKU9	Beta coronavirus	Nobecovirus
FJ647226.1	MHV	Beta coronavirus	Enbecovirus
AY884001	HCoV_HKU1	Beta coronavirus	Enbecovirus
KM349742.1	ChRCoV_HKU24	Beta coronavirus	Enbecovirus
KF923903	HCoV OC43	Beta coronavirus	Enbecovirus
KT368891.1	camel HKU23	Beta coronavirus	Enbecovirus

Table S2. List of sequences of 106 representative SARS-CoV-2 variants of concern (VOCs) and variants of interest (VOIs) sequences used in this analysis.

Accession	Pangolin	Length	Protein	Collection_Date
WKU88364	XBB.1.16	7093	ORF1ab polyprotein	2023/7/3
WKU88365	XBB.1.16	4402	ORF1a polyprotein	2023/7/3
WKU88366	XBB.1.16	1269	surface glycoprotein	2023/7/3
WKU88367	XBB.1.16	274	ORF3a protein	2023/7/3
WKU88368	XBB.1.16	75	envelope protein	2023/7/3
WKU88369	XBB.1.16	222	membrane glycoprotein	2023/7/3
WKU88370	XBB.1.16	61	ORF6 protein	2023/7/3
WKU88371	XBB.1.16	121	ORF7a protein	2023/7/3
WKU88372	XBB.1.16	43	ORF7b protein	2023/7/3
WKU88373	XBB.1.16	416	nucleocapsid phosphoprotein	2023/7/3
WKU88374	XBB.1.16	38	ORF10 protein	2023/7/3
WKU88375	XBB.1.5	7093	ORF1ab polyprotein	2023/7/4
WKU88376	XBB.1.5	4402	ORF1a polyprotein	2023/7/4
WKU88377	XBB.1.5	1269	surface glycoprotein	2023/7/4
WKU88378	XBB.1.5	275	ORF3a protein	2023/7/4
WKU88379	XBB.1.5	75	envelope protein	2023/7/4
WKU88380	XBB.1.5	222	membrane glycoprotein	2023/7/4
WKU88381	XBB.1.5	61	ORF6 protein	2023/7/4
WKU88382	XBB.1.5	121	ORF7a protein	2023/7/4
WKU88383	XBB.1.5	43	ORF7b protein	2023/7/4
WKU88384	XBB.1.5	416	nucleocapsid phosphoprotein	2023/7/4
WKU88385	XBB.1.5	37	ORF10 protein	2023/7/4
WKU88431	XBB.1.16.11	7093	ORF1ab polyprotein	2023/7/5
WKU88432	XBB.1.16.11	4402	ORF1a polyprotein	2023/7/5
WKU88433	XBB.1.16.11	1269	surface glycoprotein	2023/7/5
WKU88434	XBB.1.16.11	275	ORF3a protein	2023/7/5
WKU88435	XBB.1.16.11	75	envelope protein	2023/7/5
WKU88436	XBB.1.16.11	222	membrane glycoprotein	2023/7/5
WKU88437	XBB.1.16.11	61	ORF6 protein	2023/7/5
WKU88438	XBB.1.16.11	121	ORF7a protein	2023/7/5
WKU88439	XBB.1.16.11	43	ORF7b protein	2023/7/5
WKU88440	XBB.1.16.11	416	nucleocapsid phosphoprotein	2023/7/5
WKU88441	XBB.1.16.11	38	ORF10 protein	2023/7/5
WKU88485	XBB.2.3.2	7093	ORF1ab polyprotein	2023/7/5
WKU88486	XBB.2.3.2	4402	ORF1a polyprotein	2023/7/5
WKU88487	XBB.2.3.2	1269	surface glycoprotein	2023/7/5
WKU88488	XBB.2.3.2	275	ORF3a protein	2023/7/5
WKU88489	XBB.2.3.2	75	envelope protein	2023/7/5
WKU88490	XBB.2.3.2	222	membrane glycoprotein	2023/7/5

WKU88491	XBB.2.3.2	61	ORF6 protein	2023/7/5
WKU88492	XBB.2.3.2	121	ORF7a protein	2023/7/5
WKU88493	XBB.2.3.2	43	ORF7b protein	2023/7/5
WKU88494	XBB.2.3.2	121	ORF8 protein	2023/7/5
WKU88495	XBB.2.3.2	416	nucleocapsid phosphoprotein	2023/7/5
WKU88496	XBB.2.3.2	38	ORF10 protein	2023/7/5
WKU88520	XBB.1.16.6	7093	ORF1ab polyprotein	2023/7/5
WKU88521	XBB.1.16.6	4402	ORF1a polyprotein	2023/7/5
WKU88522	XBB.1.16.6	1269	surface glycoprotein	2023/7/5
WKU88523	XBB.1.16.6	275	ORF3a protein	2023/7/5
WKU88524	XBB.1.16.6	75	envelope protein	2023/7/5
WKU88525	XBB.1.16.6	222	membrane glycoprotein	2023/7/5
WKU88526	XBB.1.16.6	61	ORF6 protein	2023/7/5
WKU88527	XBB.1.16.6	121	ORF7a protein	2023/7/5
WKU88528	XBB.1.16.6	43	ORF7b protein	2023/7/5
WKU88529	XBB.1.16.6	416	nucleocapsid phosphoprotein	2023/7/5
WKU88530	XBB.1.16.6	37	ORF10 protein	2023/7/5
WKU88630	XBB.1.16.1	7093	ORF1ab polyprotein	2023/7/6
WKU88631	XBB.1.16.1	4402	ORF1a polyprotein	2023/7/6
WKU88632	XBB.1.16.1	1269	surface glycoprotein	2023/7/6
WKU88633	XBB.1.16.1	275	ORF3a protein	2023/7/6
WKU88634	XBB.1.16.1	75	envelope protein	2023/7/6
WKU88635	XBB.1.16.1	222	membrane glycoprotein	2023/7/6
WKU88636	XBB.1.16.1	61	ORF6 protein	2023/7/6
WKU88637	XBB.1.16.1	121	ORF7a protein	2023/7/6
WKU88638	XBB.1.16.1	43	ORF7b protein	2023/7/6
WKU88639	XBB.1.16.1	416	nucleocapsid phosphoprotein	2023/7/6
WKU88640	XBB.1.16.1	38	ORF10 protein	2023/7/6
WKU88750	XBB.2.3.12	7093	ORF1ab polyprotein	2023/7/7
WKU88751	XBB.2.3.12	4402	ORF1a polyprotein	2023/7/7
WKU88752	XBB.2.3.12	1269	surface glycoprotein	2023/7/7
WKU88753	XBB.2.3.12	275	ORF3a protein	2023/7/7
WKU88754	XBB.2.3.12	75	envelope protein	2023/7/7
WKU88755	XBB.2.3.12	222	membrane glycoprotein	2023/7/7
WKU88756	XBB.2.3.12	61	ORF6 protein	2023/7/7
WKU88757	XBB.2.3.12	121	ORF7a protein	2023/7/7
WKU88758	XBB.2.3.12	43	ORF7b protein	2023/7/7
WKU88759	XBB.2.3.12	121	ORF8 protein	2023/7/7
WKU88760	XBB.2.3.12	416	nucleocapsid phosphoprotein	2023/7/7
WKU88859	XBB.2.3.3	7093	ORF1ab polyprotein	2023/7/8
WKU88860	XBB.2.3.3	4402	ORF1a polyprotein	2023/7/8
WKU88861	XBB.2.3.3	1269	surface glycoprotein	2023/7/8

WKU88862	XBB.2.3.3	275	ORF3a protein	2023/7/8
WKU88863	XBB.2.3.3	75	envelope protein	2023/7/8
WKU88864	XBB.2.3.3	222	membrane glycoprotein	2023/7/8
WKU88865	XBB.2.3.3	61	ORF6 protein	2023/7/8
WKU88866	XBB.2.3.3	121	ORF7a protein	2023/7/8
WKU88867	XBB.2.3.3	121	ORF8 protein	2023/7/8
WKU88868	XBB.2.3.3	416	nucleocapsid phosphoprotein	2023/7/8
WKU88869	XBB.2.3.3	38	ORF10 protein	2023/7/8
WKU89036	XBB.1.9.2	7093	ORF1ab polyprotein	2023/7/10
WKU89037	XBB.1.9.2	4402	ORF1a polyprotein	2023/7/10
WKU89038	XBB.1.9.2	1269	surface glycoprotein	2023/7/10
WKU89039	XBB.1.9.2	275	ORF3a protein	2023/7/10
WKU89040	XBB.1.9.2	75	envelope protein	2023/7/10
WKU89041	XBB.1.9.2	222	membrane glycoprotein	2023/7/10
WKU89042	XBB.1.9.2	61	ORF6 protein	2023/7/10
WKU89043	XBB.1.9.2	121	ORF7a protein	2023/7/10
WKU89044	XBB.1.9.2	43	ORF7b protein	2023/7/10
WKU89045	XBB.1.9.2	416	nucleocapsid phosphoprotein	2023/7/10
WKU89046	XBB.1.9.2	38	ORF10 protein	2023/7/10
WKU89047	FL.25	7093	ORF1ab polyprotein	2023/7/10
WKU89048	FL.25	4402	ORF1a polyprotein	2023/7/10
WKU89049	FL.25	1269	surface glycoprotein	2023/7/10
WKU89050	FL.25	275	ORF3a protein	2023/7/10
WKU89051	FL.25	75	envelope protein	2023/7/10
WKU89052	FL.25	222	membrane glycoprotein	2023/7/10
WKU89053	FL.25	61	ORF6 protein	2023/7/10
WKU89054	FL.25	121	ORF7a protein	2023/7/10
WKU89055	FL.25	43	ORF7b protein	2023/7/10
WKU89056	FL.25	416	nucleocapsid phosphoprotein	2023/7/10
WKU89089	GY.5	7093	ORF1ab polyprotein	2023/7/10
WKU89090	GY.5	4402	ORF1a polyprotein	2023/7/10
WKU89091	GY.5	1269	surface glycoprotein	2023/7/10
WKU89092	GY.5	275	ORF3a protein	2023/7/10
WKU89093	GY.5	75	envelope protein	2023/7/10
WKU89094	GY.5	222	membrane glycoprotein	2023/7/10
WKU89095	GY.5	61	ORF6 protein	2023/7/10
WKU89096	GY.5	121	ORF7a protein	2023/7/10
WKU89097	GY.5	43	ORF7b protein	2023/7/10
WKU89098	GY.5	416	nucleocapsid phosphoprotein	2023/7/10
WKU89099	GY.5	38	ORF10 protein	2023/7/10
WKU89569	XBB.2.3	7093	ORF1ab polyprotein	2023/7/12
WKU89570	XBB.2.3	4402	ORF1a polyprotein	2023/7/12

WKU89571	XBB.2.3	1268	surface glycoprotein	2023/7/12
WKU89572	XBB.2.3	275	ORF3a protein	2023/7/12
WKU89573	XBB.2.3	75	envelope protein	2023/7/12
WKU89574	XBB.2.3	222	membrane glycoprotein	2023/7/12
WKU89575	XBB.2.3	61	ORF6 protein	2023/7/12
WKU89576	XBB.2.3	43	ORF7b protein	2023/7/12
WKU89577	XBB.2.3	121	ORF8 protein	2023/7/12
WKU89578	XBB.2.3	416	nucleocapsid phosphoprotein	2023/7/12
WKU89579	XBB.2.3	37	ORF10 protein	2023/7/12
WKU89580	XBB.1.5.7	7093	ORF1ab polyprotein	2023/7/12
WKU89581	XBB.1.5.7	4402	ORF1a polyprotein	2023/7/12
WKU89582	XBB.1.5.7	1269	surface glycoprotein	2023/7/12
WKU89583	XBB.1.5.7	275	ORF3a protein	2023/7/12
WKU89584	XBB.1.5.7	75	envelope protein	2023/7/12
WKU89585	XBB.1.5.7	222	membrane glycoprotein	2023/7/12
WKU89586	XBB.1.5.7	61	ORF6 protein	2023/7/12
WKU89587	XBB.1.5.7	121	ORF7a protein	2023/7/12
WKU89588	XBB.1.5.7	43	ORF7b protein	2023/7/12
WKU89589	XBB.1.5.7	416	nucleocapsid phosphoprotein	2023/7/12
WKU89590	XBB.1.5.7	38	ORF10 protein	2023/7/12
WKU89659	CH.1.1.15	7093	ORF1ab polyprotein	2023/7/12
WKU89660	CH.1.1.15	4402	ORF1a polyprotein	2023/7/12
WKU89661	CH.1.1.15	1270	surface glycoprotein	2023/7/12
WKU89662	CH.1.1.15	275	ORF3a protein	2023/7/12
WKU89663	CH.1.1.15	75	envelope protein	2023/7/12
WKU89664	CH.1.1.15	222	membrane glycoprotein	2023/7/12
WKU89665	CH.1.1.15	61	ORF6 protein	2023/7/12
WKU89666	CH.1.1.15	121	ORF7a protein	2023/7/12
WKU89667	CH.1.1.15	43	ORF7b protein	2023/7/12
WKU89668	CH.1.1.15	121	ORF8 protein	2023/7/12
WKU89669	CH.1.1.15	401	nucleocapsid phosphoprotein	2023/7/12
WKU89976	GZ.1	7093	ORF1ab polyprotein	2023/7/13
WKU89977	GZ.1	4402	ORF1a polyprotein	2023/7/13
WKU89978	GZ.1	1269	surface glycoprotein	2023/7/13
WKU89979	GZ.1	275	ORF3a protein	2023/7/13
WKU89980	GZ.1	75	envelope protein	2023/7/13
WKU89981	GZ.1	222	membrane glycoprotein	2023/7/13
WKU89982	GZ.1	61	ORF6 protein	2023/7/13
WKU89983	GZ.1	121	ORF7a protein	2023/7/13
WKU89984	GZ.1	43	ORF7b protein	2023/7/13
WKU89985	GZ.1	121	ORF8 protein	2023/7/13
WKU89986	GZ.1	416	nucleocapsid phosphoprotein	2023/7/13

WKU89987	GZ.1	38	ORF10 protein	2023/7/13
WKU90042	XBB.1.5.49	7093	ORF1ab polyprotein	2023/7/14
WKU90043	XBB.1.5.49	4402	ORF1a polyprotein	2023/7/14
WKU90044	XBB.1.5.49	1269	surface glycoprotein	2023/7/14
WKU90045	XBB.1.5.49	275	ORF3a protein	2023/7/14
WKU90046	XBB.1.5.49	75	envelope protein	2023/7/14
WKU90047	XBB.1.5.49	222	membrane glycoprotein	2023/7/14
WKU90048	XBB.1.5.49	61	ORF6 protein	2023/7/14
WKU90049	XBB.1.5.49	121	ORF7a protein	2023/7/14
WKU90050	XBB.1.5.49	43	ORF7b protein	2023/7/14
WKU90051	XBB.1.5.49	416	nucleocapsid phosphoprotein	2023/7/14
WKU90052	XBB.1.5.49	38	ORF10 protein	2023/7/14
WKU90076	XBB.2.3.8	7093	ORF1ab polyprotein	2023/7/14
WKU90077	XBB.2.3.8	4402	ORF1a polyprotein	2023/7/14
WKU90078	XBB.2.3.8	1269	surface glycoprotein	2023/7/14
WKU90079	XBB.2.3.8	275	ORF3a protein	2023/7/14
WKU90080	XBB.2.3.8	75	envelope protein	2023/7/14
WKU90081	XBB.2.3.8	222	membrane glycoprotein	2023/7/14
WKU90082	XBB.2.3.8	61	ORF6 protein	2023/7/14
WKU90083	XBB.2.3.8	121	ORF7a protein	2023/7/14
WKU90084	XBB.2.3.8	43	ORF7b protein	2023/7/14
WKU90085	XBB.2.3.8	121	ORF8 protein	2023/7/14
WKU90086	XBB.2.3.8	416	nucleocapsid phosphoprotein	2023/7/14
WKU90087	XBB.2.3.8	38	ORF10 protein	2023/7/14
WKU90208	XBB.1.16.8	7093	ORF1ab polyprotein	2023/7/14
WKU90209	XBB.1.16.8	4402	ORF1a polyprotein	2023/7/14
WKU90210	XBB.1.16.8	1269	surface glycoprotein	2023/7/14
WKU90211	XBB.1.16.8	275	ORF3a protein	2023/7/14
WKU90212	XBB.1.16.8	75	envelope protein	2023/7/14
WKU90213	XBB.1.16.8	222	membrane glycoprotein	2023/7/14
WKU90214	XBB.1.16.8	61	ORF6 protein	2023/7/14
WKU90215	XBB.1.16.8	121	ORF7a protein	2023/7/14
WKU90216	XBB.1.16.8	43	ORF7b protein	2023/7/14
WKU90217	XBB.1.16.8	416	nucleocapsid phosphoprotein	2023/7/14
WKU90218	XBB.1.16.8	17	ORF10 protein	2023/7/14
WKU90546	BA.5.2	7093	ORF1ab polyprotein	2022/7/6
WKU90547	BA.5.2	4402	ORF1a polyprotein	2022/7/6
WKU90548	BA.5.2	1268	surface glycoprotein	2022/7/6
WKU90549	BA.5.2	275	ORF3a protein	2022/7/6
WKU90550	BA.5.2	75	envelope protein	2022/7/6
WKU90551	BA.5.2	222	membrane glycoprotein	2022/7/6
WKU90552	BA.5.2	61	ORF6 protein	2022/7/6

WKU90553	BA.5.2	121	ORF7a protein	2022/7/6
WKU90554	BA.5.2	43	ORF7b protein	2022/7/6
WKU90555	BA.5.2	121	ORF8 protein	2022/7/6
WKU90556	BA.5.2	416	nucleocapsid phosphoprotein	2022/7/6
WKU90557	BA.5.2	38	ORF10 protein	2022/7/6
WKU90558	BA.5.2.1	7093	ORF1ab polyprotein	2022/7/7
WKU90559	BA.5.2.1	4402	ORF1a polyprotein	2022/7/7
WKU90560	BA.5.2.1	1268	surface glycoprotein	2022/7/7
WKU90561	BA.5.2.1	275	ORF3a protein	2022/7/7
WKU90562	BA.5.2.1	75	envelope protein	2022/7/7
WKU90563	BA.5.2.1	222	membrane glycoprotein	2022/7/7
WKU90564	BA.5.2.1	61	ORF6 protein	2022/7/7
WKU90565	BA.5.2.1	121	ORF7a protein	2022/7/7
WKU90566	BA.5.2.1	121	ORF8 protein	2022/7/7
WKU90567	BA.5.2.1	416	nucleocapsid phosphoprotein	2022/7/7
WKU90568	BA.5.2.1	38	ORF10 protein	2022/7/7
WKU90737	BA.2.12.1	7066	ORF1ab polyprotein	2022/7/11
WKU90738	BA.2.12.1	4375	ORF1a polyprotein	2022/7/11
WKU90739	BA.2.12.1	1270	surface glycoprotein	2022/7/11
WKU90740	BA.2.12.1	275	ORF3a protein	2022/7/11
WKU90741	BA.2.12.1	75	envelope protein	2022/7/11
WKU90742	BA.2.12.1	222	membrane glycoprotein	2022/7/11
WKU90743	BA.2.12.1	61	ORF6 protein	2022/7/11
WKU90744	BA.2.12.1	121	ORF7a protein	2022/7/11
WKU90745	BA.2.12.1	43	ORF7b protein	2022/7/11
WKU90746	BA.2.12.1	121	ORF8 protein	2022/7/11
WKU90747	BA.2.12.1	410	nucleocapsid phosphoprotein	2022/7/11
WKU90748	BA.2.12.1	35	ORF10 protein	2022/7/11
WKU90831	BA.5.1	7093	ORF1ab polyprotein	2022/7/7
WKU90832	BA.5.1	4402	ORF1a polyprotein	2022/7/7
WKU90833	BA.5.1	1268	surface glycoprotein	2022/7/7
WKU90834	BA.5.1	275	ORF3a protein	2022/7/7
WKU90835	BA.5.1	75	envelope protein	2022/7/7
WKU90836	BA.5.1	222	membrane glycoprotein	2022/7/7
WKU90837	BA.5.1	61	ORF6 protein	2022/7/7
WKU90838	BA.5.1	121	ORF7a protein	2022/7/7
WKU90839	BA.5.1	43	ORF7b protein	2022/7/7
WKU90840	BA.5.1	121	ORF8 protein	2022/7/7
WKU90841	BA.5.1	416	nucleocapsid phosphoprotein	2022/7/7
WKU90842	BA.5.1	38	ORF10 protein	2022/7/7
WKU91250	BA.2.9	7093	ORF1ab polyprotein	2022/7/7
WKU91251	BA.2.9	4402	ORF1a polyprotein	2022/7/7

WKU91252	BA.2.9	1270	surface glycoprotein	2022/7/7
WKU91253	BA.2.9	275	ORF3a protein	2022/7/7
WKU91254	BA.2.9	75	envelope protein	2022/7/7
WKU91255	BA.2.9	222	membrane glycoprotein	2022/7/7
WKU91256	BA.2.9	61	ORF6 protein	2022/7/7
WKU91257	BA.2.9	121	ORF7a protein	2022/7/7
WKU91258	BA.2.9	43	ORF7b protein	2022/7/7
WKU91259	BA.2.9	121	ORF8 protein	2022/7/7
WKU91260	BA.2.9	416	nucleocapsid phosphoprotein	2022/7/7
WKU91261	BA.2.9	38	ORF10 protein	2022/7/7
WKU91477	BA.2	7066	ORF1ab polyprotein	2022/6/8
WKU91478	BA.2	4375	ORF1a polyprotein	2022/6/8
WKU91479	BA.2	1270	surface glycoprotein	2022/6/8
WKU91480	BA.2	275	ORF3a protein	2022/6/8
WKU91481	BA.2	75	envelope protein	2022/6/8
WKU91482	BA.2	222	membrane glycoprotein	2022/6/8
WKU91483	BA.2	61	ORF6 protein	2022/6/8
WKU91484	BA.2	121	ORF7a protein	2022/6/8
WKU91485	BA.2	43	ORF7b protein	2022/6/8
WKU91486	BA.2	121	ORF8 protein	2022/6/8
WKU91487	BA.2	416	nucleocapsid phosphoprotein	2022/6/8
WKU91488	BA.2	38	ORF10 protein	2022/6/8
WKV28504	XBB.2.3.11	7093	ORF1ab polyprotein	2023/6/25
WKV28505	XBB.2.3.11	4402	ORF1a polyprotein	2023/6/25
WKV28506	XBB.2.3.11	1269	surface glycoprotein	2023/6/25
WKV28507	XBB.2.3.11	275	ORF3a protein	2023/6/25
WKV28508	XBB.2.3.11	75	envelope protein	2023/6/25
WKV28509	XBB.2.3.11	222	membrane glycoprotein	2023/6/25
WKV28510	XBB.2.3.11	61	ORF6 protein	2023/6/25
WKV28511	XBB.2.3.11	121	ORF7a protein	2023/6/25
WKV28512	XBB.2.3.11	43	ORF7b protein	2023/6/25
WKV28513	XBB.2.3.11	121	ORF8 protein	2023/6/25
WKV28514	XBB.2.3.11	416	nucleocapsid phosphoprotein	2023/6/25
WKV28515	XBB.2.3.11	38	ORF10 protein	2023/6/25
WKV30480	XBB.1.5.32	7093	ORF1ab polyprotein	2023/5/18
WKV30481	XBB.1.5.32	4402	ORF1a polyprotein	2023/5/18
WKV30482	XBB.1.5.32	1269	surface glycoprotein	2023/5/18
WKV30483	XBB.1.5.32	275	ORF3a protein	2023/5/18
WKV30484	XBB.1.5.32	75	envelope protein	2023/5/18
WKV30485	XBB.1.5.32	222	membrane glycoprotein	2023/5/18
WKV30486	XBB.1.5.32	61	ORF6 protein	2023/5/18
WKV30487	XBB.1.5.32	121	ORF7a protein	2023/5/18

WKV30488	XBB.1.5.32	42	ORF7b protein	2023/5/18
WKV30489	XBB.1.5.32	416	nucleocapsid phosphoprotein	2023/5/18
WKV30490	XBB.1.5.32	38	ORF10 protein	2023/5/18
WKV30580	XBB.1.5.1	7093	ORF1ab polyprotein	2023/5/22
WKV30581	XBB.1.5.1	4402	ORF1a polyprotein	2023/5/22
WKV30582	XBB.1.5.1	1269	surface glycoprotein	2023/5/22
WKV30583	XBB.1.5.1	275	ORF3a protein	2023/5/22
WKV30584	XBB.1.5.1	75	envelope protein	2023/5/22
WKV30585	XBB.1.5.1	222	membrane glycoprotein	2023/5/22
WKV30586	XBB.1.5.1	61	ORF6 protein	2023/5/22
WKV30587	XBB.1.5.1	121	ORF7a protein	2023/5/22
WKV30588	XBB.1.5.1	43	ORF7b protein	2023/5/22
WKV30589	XBB.1.5.1	416	nucleocapsid phosphoprotein	2023/5/22
WKV30590	XBB.1.5.1	38	ORF10 protein	2023/5/22
WKV30711	XBB.1.5.17	7093	ORF1ab polyprotein	2023/5/18
WKV30712	XBB.1.5.17	4402	ORF1a polyprotein	2023/5/18
WKV30713	XBB.1.5.17	1269	surface glycoprotein	2023/5/18
WKV30714	XBB.1.5.17	275	ORF3a protein	2023/5/18
WKV30715	XBB.1.5.17	75	envelope protein	2023/5/18
WKV30716	XBB.1.5.17	222	membrane glycoprotein	2023/5/18
WKV30717	XBB.1.5.17	61	ORF6 protein	2023/5/18
WKV30718	XBB.1.5.17	121	ORF7a protein	2023/5/18
WKV30719	XBB.1.5.17	43	ORF7b protein	2023/5/18
WKV30720	XBB.1.5.17	416	nucleocapsid phosphoprotein	2023/5/18
WKV30721	XBB.1.5.17	38	ORF10 protein	2023/5/18
WKV30755	XBB.1.5.15	7093	ORF1ab polyprotein	2023/5/20
WKV30756	XBB.1.5.15	4402	ORF1a polyprotein	2023/5/20
WKV30757	XBB.1.5.15	1269	surface glycoprotein	2023/5/20
WKV30758	XBB.1.5.15	275	ORF3a protein	2023/5/20
WKV30759	XBB.1.5.15	75	envelope protein	2023/5/20
WKV30760	XBB.1.5.15	222	membrane glycoprotein	2023/5/20
WKV30761	XBB.1.5.15	61	ORF6 protein	2023/5/20
WKV30762	XBB.1.5.15	121	ORF7a protein	2023/5/20
WKV30763	XBB.1.5.15	43	ORF7b protein	2023/5/20
WKV30764	XBB.1.5.15	416	nucleocapsid phosphoprotein	2023/5/20
WKV30765	XBB.1.5.15	38	ORF10 protein	2023/5/20
WKV31017	XBB.1.5.13	7093	ORF1ab polyprotein	2023/5/18
WKV31018	XBB.1.5.13	4402	ORF1a polyprotein	2023/5/18
WKV31019	XBB.1.5.13	1269	surface glycoprotein	2023/5/18
WKV31020	XBB.1.5.13	275	ORF3a protein	2023/5/18
WKV31021	XBB.1.5.13	75	envelope protein	2023/5/18
WKV31022	XBB.1.5.13	222	membrane glycoprotein	2023/5/18

WKV31023	XBB.1.5.13	61	ORF6 protein	2023/5/18
WKV31024	XBB.1.5.13	121	ORF7a protein	2023/5/18
WKV31025	XBB.1.5.13	43	ORF7b protein	2023/5/18
WKV31026	XBB.1.5.13	416	nucleocapsid phosphoprotein	2023/5/18
WKV31027	XBB.1.5.13	38	ORF10 protein	2023/5/18
WKV35613	XBB.1.16.5	7093	ORF1ab polyprotein	2023/7/9
WKV35614	XBB.1.16.5	4402	ORF1a polyprotein	2023/7/9
WKV35615	XBB.1.16.5	1269	surface glycoprotein	2023/7/9
WKV35616	XBB.1.16.5	275	ORF3a protein	2023/7/9
WKV35617	XBB.1.16.5	75	envelope protein	2023/7/9
WKV35618	XBB.1.16.5	222	membrane glycoprotein	2023/7/9
WKV35619	XBB.1.16.5	61	ORF6 protein	2023/7/9
WKV35620	XBB.1.16.5	121	ORF7a protein	2023/7/9
WKV35621	XBB.1.16.5	43	ORF7b protein	2023/7/9
WKV35622	XBB.1.16.5	416	nucleocapsid phosphoprotein	2023/7/9
WKV35623	XBB.1.16.5	38	ORF10 protein	2023/7/9
WKV36782	CH.1.1.2	7093	ORF1ab polyprotein	2023/6/14
WKV36783	CH.1.1.2	4402	ORF1a polyprotein	2023/6/14
WKV36784	CH.1.1.2	1270	surface glycoprotein	2023/6/14
WKV36785	CH.1.1.2	275	ORF3a protein	2023/6/14
WKV36786	CH.1.1.2	75	envelope protein	2023/6/14
WKV36787	CH.1.1.2	222	membrane glycoprotein	2023/6/14
WKV36788	CH.1.1.2	61	ORF6 protein	2023/6/14
WKV36789	CH.1.1.2	121	ORF7a protein	2023/6/14
WKV36790	CH.1.1.2	43	ORF7b protein	2023/6/14
WKV36791	CH.1.1.2	121	ORF8 protein	2023/6/14
WKV36792	CH.1.1.2	416	nucleocapsid phosphoprotein	2023/6/14
WKV36793	CH.1.1.2	38	ORF10 protein	2023/6/14
WKU09913	XBB.1.5.18	7093	ORF1ab polyprotein	2023/7/3
WKU09914	XBB.1.5.18	4402	ORF1a polyprotein	2023/7/3
WKU09915	XBB.1.5.18	1269	surface glycoprotein	2023/7/3
WKU09916	XBB.1.5.18	275	ORF3a protein	2023/7/3
WKU09917	XBB.1.5.18	75	envelope protein	2023/7/3
WKU09918	XBB.1.5.18	222	membrane glycoprotein	2023/7/3
WKU09919	XBB.1.5.18	61	ORF6 protein	2023/7/3
WKU09920	XBB.1.5.18	121	ORF7a protein	2023/7/3
WKU09921	XBB.1.5.18	43	ORF7b protein	2023/7/3
WKU09922	XBB.1.5.18	416	nucleocapsid phosphoprotein	2023/7/3
WKU09923	XBB.1.5.18	38	ORF10 protein	2023/7/3
WKU11379	XBB.1.16.3	7093	ORF1ab polyprotein	2023/7/6
WKU11380	XBB.1.16.3	4402	ORF1a polyprotein	2023/7/6
WKU11381	XBB.1.16.3	1268	surface glycoprotein	2023/7/6

WKU11382	XBB.1.16.3	275	ORF3a protein	2023/7/6
WKU11383	XBB.1.16.3	75	envelope protein	2023/7/6
WKU11384	XBB.1.16.3	222	membrane glycoprotein	2023/7/6
WKU11385	XBB.1.16.3	61	ORF6 protein	2023/7/6
WKU11386	XBB.1.16.3	121	ORF7a protein	2023/7/6
WKU11387	XBB.1.16.3	43	ORF7b protein	2023/7/6
WKU11388	XBB.1.16.3	416	nucleocapsid phosphoprotein	2023/7/6
WKU11389	XBB.1.16.3	38	ORF10 protein	2023/7/6
WKU11559	XBB.2.3.5	7093	ORF1ab polyprotein	2023/7/6
WKU11560	XBB.2.3.5	4402	ORF1a polyprotein	2023/7/6
WKU11561	XBB.2.3.5	1269	surface glycoprotein	2023/7/6
WKU11562	XBB.2.3.5	275	ORF3a protein	2023/7/6
WKU11563	XBB.2.3.5	75	envelope protein	2023/7/6
WKU11564	XBB.2.3.5	222	membrane glycoprotein	2023/7/6
WKU11565	XBB.2.3.5	61	ORF6 protein	2023/7/6
WKU11566	XBB.2.3.5	121	ORF7a protein	2023/7/6
WKU11567	XBB.2.3.5	43	ORF7b protein	2023/7/6
WKU11568	XBB.2.3.5	121	ORF8 protein	2023/7/6
WKU11569	XBB.2.3.5	416	nucleocapsid phosphoprotein	2023/7/6
WKU11570	XBB.2.3.5	38	ORF10 protein	2023/7/6
WKU61286	XBB.1.16.9	7093	ORF1ab polyprotein	2023/6/29
WKU61287	XBB.1.16.9	4402	ORF1a polyprotein	2023/6/29
WKU61288	XBB.1.16.9	1269	surface glycoprotein	2023/6/29
WKU61289	XBB.1.16.9	275	ORF3a protein	2023/6/29
WKU61290	XBB.1.16.9	75	envelope protein	2023/6/29
WKU61291	XBB.1.16.9	222	membrane glycoprotein	2023/6/29
WKU61292	XBB.1.16.9	61	ORF6 protein	2023/6/29
WKU61293	XBB.1.16.9	121	ORF7a protein	2023/6/29
WKU61294	XBB.1.16.9	43	ORF7b protein	2023/6/29
WKU61295	XBB.1.16.9	416	nucleocapsid phosphoprotein	2023/6/29
WKU61296	XBB.1.16.9	38	ORF10 protein	2023/6/29
WKU88022	XBB.1.16.10	7093	ORF1ab polyprotein	2023/6/15
WKU88023	XBB.1.16.10	4402	ORF1a polyprotein	2023/6/15
WKU88024	XBB.1.16.10	1269	surface glycoprotein	2023/6/15
WKU88025	XBB.1.16.10	275	ORF3a protein	2023/6/15
WKU88026	XBB.1.16.10	75	envelope protein	2023/6/15
WKU88027	XBB.1.16.10	222	membrane glycoprotein	2023/6/15
WKU88028	XBB.1.16.10	61	ORF6 protein	2023/6/15
WKU88029	XBB.1.16.10	121	ORF7a protein	2023/6/15
WKU88030	XBB.1.16.10	43	ORF7b protein	2023/6/15
WKU88031	XBB.1.16.10	416	nucleocapsid phosphoprotein	2023/6/15
WKU88032	XBB.1.16.10	38	ORF10 protein	2023/6/15

WKR36895	BQ.1.1	7093	ORF1ab polyprotein	2023/7/5
WKR36896	BQ.1.1	4402	ORF1a polyprotein	2023/7/5
WKR36897	BQ.1.1	1268	surface glycoprotein	2023/7/5
WKR36898	BQ.1.1	275	ORF3a protein	2023/7/5
WKR36899	BQ.1.1	75	envelope protein	2023/7/5
WKR36900	BQ.1.1	222	membrane glycoprotein	2023/7/5
WKR36901	BQ.1.1	61	ORF6 protein	2023/7/5
WKR36902	BQ.1.1	121	ORF7a protein	2023/7/5
WKR36903	BQ.1.1	43	ORF7b protein	2023/7/5
WKR36904	BQ.1.1	121	ORF8 protein	2023/7/5
WKR36905	BQ.1.1	416	nucleocapsid phosphoprotein	2023/7/5
WKR36906	BQ.1.1	38	ORF10 protein	2023/7/5
WKS75852	XBB.1.16.4	7093	ORF1ab polyprotein	2023/7/2
WKS75853	XBB.1.16.4	4402	ORF1a polyprotein	2023/7/2
WKS75854	XBB.1.16.4	1269	surface glycoprotein	2023/7/2
WKS75855	XBB.1.16.4	275	ORF3a protein	2023/7/2
WKS75856	XBB.1.16.4	75	envelope protein	2023/7/2
WKS75857	XBB.1.16.4	222	membrane glycoprotein	2023/7/2
WKS75858	XBB.1.16.4	61	ORF6 protein	2023/7/2
WKS75859	XBB.1.16.4	121	ORF7a protein	2023/7/2
WKS75860	XBB.1.16.4	43	ORF7b protein	2023/7/2
WKS75861	XBB.1.16.4	416	nucleocapsid phosphoprotein	2023/7/2
WKS75862	XBB.1.16.4	38	ORF10 protein	2023/7/2
WKT26071	XBB.2.3.1	7093	ORF1ab polyprotein	2023/6/14
WKT26072	XBB.2.3.1	4402	ORF1a polyprotein	2023/6/14
WKT26073	XBB.2.3.1	1269	surface glycoprotein	2023/6/14
WKT26074	XBB.2.3.1	275	ORF3a protein	2023/6/14
WKT26075	XBB.2.3.1	75	envelope protein	2023/6/14
WKT26076	XBB.2.3.1	222	membrane glycoprotein	2023/6/14
WKT26077	XBB.2.3.1	61	ORF6 protein	2023/6/14
WKT26078	XBB.2.3.1	121	ORF7a protein	2023/6/14
WKT26079	XBB.2.3.1	43	ORF7b protein	2023/6/14
WKT26080	XBB.2.3.1	121	ORF8 protein	2023/6/14
WKT26081	XBB.2.3.1	416	nucleocapsid phosphoprotein	2023/6/14
WKT26082	XBB.2.3.1	38	ORF10 protein	2023/6/14
WKN81912	B.1.2	7096	ORF1ab polyprotein	2020/12/26
WKN81913	B.1.2	4405	ORF1a polyprotein	2020/12/26
WKN81914	B.1.2	1273	surface glycoprotein	2020/12/26
WKN81915	B.1.2	275	ORF3a protein	2020/12/26
WKN81916	B.1.2	75	envelope protein	2020/12/26
WKN81917	B.1.2	222	membrane glycoprotein	2020/12/26
WKN81918	B.1.2	61	ORF6 protein	2020/12/26

WKN81919	B.1.2	121	ORF7a protein	2020/12/26
WKN81920	B.1.2	43	ORF7b protein	2020/12/26
WKN81921	B.1.2	121	ORF8 protein	2020/12/26
WKN81922	B.1.2	419	nucleocapsid phosphoprotein	2020/12/26
WKN81923	B.1.2	38	ORF10 protein	2020/12/26
WKN82137	AY.25	7096	ORF1ab polyprotein	2021/8/16
WKN82138	AY.25	4405	ORF1a polyprotein	2021/8/16
WKN82139	AY.25	1271	surface glycoprotein	2021/8/16
WKN82140	AY.25	275	ORF3a protein	2021/8/16
WKN82141	AY.25	75	envelope protein	2021/8/16
WKN82142	AY.25	222	membrane glycoprotein	2021/8/16
WKN82143	AY.25	61	ORF6 protein	2021/8/16
WKN82144	AY.25	121	ORF7a protein	2021/8/16
WKN82145	AY.25	43	ORF7b protein	2021/8/16
WKN82146	AY.25	119	ORF8 protein	2021/8/16
WKN82147	AY.25	419	nucleocapsid phosphoprotein	2021/8/16
WKN82148	AY.25	38	ORF10 protein	2021/8/16
WKN82172	AY.3	7096	ORF1ab polyprotein	2021/7/8
WKN82173	AY.3	4405	ORF1a polyprotein	2021/7/8
WKN82174	AY.3	1271	surface glycoprotein	2021/7/8
WKN82175	AY.3	275	ORF3a protein	2021/7/8
WKN82176	AY.3	75	envelope protein	2021/7/8
WKN82177	AY.3	222	membrane glycoprotein	2021/7/8
WKN82178	AY.3	61	ORF6 protein	2021/7/8
WKN82179	AY.3	121	ORF7a protein	2021/7/8
WKN82180	AY.3	43	ORF7b protein	2021/7/8
WKN82181	AY.3	119	ORF8 protein	2021/7/8
WKN82182	AY.3	419	nucleocapsid phosphoprotein	2021/7/8
WKN82183	AY.3	38	ORF10 protein	2021/7/8
WKN82184	AY.44	7096	ORF1ab polyprotein	2021/7/3
WKN82185	AY.44	4405	ORF1a polyprotein	2021/7/3
WKN82186	AY.44	1271	surface glycoprotein	2021/7/3
WKN82187	AY.44	275	ORF3a protein	2021/7/3
WKN82188	AY.44	75	envelope protein	2021/7/3
WKN82189	AY.44	222	membrane glycoprotein	2021/7/3
WKN82190	AY.44	61	ORF6 protein	2021/7/3
WKN82191	AY.44	121	ORF7a protein	2021/7/3
WKN82192	AY.44	43	ORF7b protein	2021/7/3
WKN82193	AY.44	119	ORF8 protein	2021/7/3
WKN82194	AY.44	419	nucleocapsid phosphoprotein	2021/7/3
WKN82195	AY.44	38	ORF10 protein	2021/7/3
WKN82220	AY.103	7096	ORF1ab polyprotein	2021/8/10

WKN82221	AY.103	4405	ORF1a polyprotein	2021/8/10
WKN82222	AY.103	1271	surface glycoprotein	2021/8/10
WKN82223	AY.103	275	ORF3a protein	2021/8/10
WKN82224	AY.103	75	envelope protein	2021/8/10
WKN82225	AY.103	222	membrane glycoprotein	2021/8/10
WKN82226	AY.103	61	ORF6 protein	2021/8/10
WKN82227	AY.103	121	ORF7a protein	2021/8/10
WKN82228	AY.103	43	ORF7b protein	2021/8/10
WKN82229	AY.103	119	ORF8 protein	2021/8/10
WKN82230	AY.103	419	nucleocapsid phosphoprotein	2021/8/10
WKN82231	AY.103	38	ORF10 protein	2021/8/10
WKN84138	AY.122	7096	ORF1ab polyprotein	2021/9/16
WKN84139	AY.122	4405	ORF1a polyprotein	2021/9/16
WKN84140	AY.122	1271	surface glycoprotein	2021/9/16
WKN84141	AY.122	275	ORF3a protein	2021/9/16
WKN84142	AY.122	75	envelope protein	2021/9/16
WKN84143	AY.122	222	membrane glycoprotein	2021/9/16
WKN84144	AY.122	61	ORF6 protein	2021/9/16
WKN84145	AY.122	121	ORF7a protein	2021/9/16
WKN84146	AY.122	43	ORF7b protein	2021/9/16
WKN84147	AY.122	119	ORF8 protein	2021/9/16
WKN84148	AY.122	419	nucleocapsid phosphoprotein	2021/9/16
WKN84149	AY.122	38	ORF10 protein	2021/9/16
WKN84220	B.1.1.7	7093	ORF1ab polyprotein	2021/2/9
WKN84221	B.1.1.7	4402	ORF1a polyprotein	2021/2/9
WKN84222	B.1.1.7	1270	surface glycoprotein	2021/2/9
WKN84223	B.1.1.7	275	ORF3a protein	2021/2/9
WKN84224	B.1.1.7	75	envelope protein	2021/2/9
WKN84225	B.1.1.7	222	membrane glycoprotein	2021/2/9
WKN84226	B.1.1.7	61	ORF6 protein	2021/2/9
WKN84227	B.1.1.7	121	ORF7a protein	2021/2/9
WKN84228	B.1.1.7	43	ORF7b protein	2021/2/9
WKN84229	B.1.1.7	419	nucleocapsid phosphoprotein	2021/2/9
WKN84230	B.1.1.7	38	ORF10 protein	2021/2/9
WKN84375	AY.100	7096	ORF1ab polyprotein	2021/8/27
WKN84376	AY.100	4405	ORF1a polyprotein	2021/8/27
WKN84377	AY.100	1271	surface glycoprotein	2021/8/27
WKN84378	AY.100	275	ORF3a protein	2021/8/27
WKN84379	AY.100	75	envelope protein	2021/8/27
WKN84380	AY.100	222	membrane glycoprotein	2021/8/27
WKN84381	AY.100	61	ORF6 protein	2021/8/27
WKN84382	AY.100	121	ORF7a protein	2021/8/27

WKN84383	AY.100	43	ORF7b protein	2021/8/27
WKN84384	AY.100	119	ORF8 protein	2021/8/27
WKN84385	AY.100	419	nucleocapsid phosphoprotein	2021/8/27
WKN84386	AY.100	38	ORF10 protein	2021/8/27
WKN84517	B.1.617.2	7096	ORF1ab polyprotein	2021/7/15
WKN84518	B.1.617.2	4405	ORF1a polyprotein	2021/7/15
WKN84519	B.1.617.2	1271	surface glycoprotein	2021/7/15
WKN84520	B.1.617.2	274	ORF3a protein	2021/7/15
WKN84521	B.1.617.2	75	envelope protein	2021/7/15
WKN84522	B.1.617.2	222	membrane glycoprotein	2021/7/15
WKN84523	B.1.617.2	61	ORF6 protein	2021/7/15
WKN84524	B.1.617.2	121	ORF7a protein	2021/7/15
WKN84525	B.1.617.2	43	ORF7b protein	2021/7/15
WKN84526	B.1.617.2	119	ORF8 protein	2021/7/15
WKN84527	B.1.617.2	419	nucleocapsid phosphoprotein	2021/7/15
WKN84528	B.1.617.2	38	ORF10 protein	2021/7/15
WKN84862	AY.25.1	7096	ORF1ab polyprotein	2021/9/20
WKN84863	AY.25.1	4405	ORF1a polyprotein	2021/9/20
WKN84864	AY.25.1	1271	surface glycoprotein	2021/9/20
WKN84865	AY.25.1	275	ORF3a protein	2021/9/20
WKN84866	AY.25.1	75	envelope protein	2021/9/20
WKN84867	AY.25.1	222	membrane glycoprotein	2021/9/20
WKN84868	AY.25.1	61	ORF6 protein	2021/9/20
WKN84869	AY.25.1	121	ORF7a protein	2021/9/20
WKN84870	AY.25.1	43	ORF7b protein	2021/9/20
WKN84871	AY.25.1	119	ORF8 protein	2021/9/20
WKN84872	AY.25.1	419	nucleocapsid phosphoprotein	2021/9/20
WKN84873	AY.25.1	38	ORF10 protein	2021/9/20
WKN88551	P.1	7093	ORF1ab polyprotein	2021/4/6
WKN88552	P.1	4402	ORF1a polyprotein	2021/4/6
WKN88553	P.1	1273	surface glycoprotein	2021/4/6
WKN88554	P.1	275	ORF3a protein	2021/4/6
WKN88555	P.1	75	envelope protein	2021/4/6
WKN88556	P.1	222	membrane glycoprotein	2021/4/6
WKN88557	P.1	61	ORF6 protein	2021/4/6
WKN88558	P.1	121	ORF7a protein	2021/4/6
WKN88559	P.1	43	ORF7b protein	2021/4/6
WKN88560	P.1	121	ORF8 protein	2021/4/6
WKN88561	P.1	419	nucleocapsid phosphoprotein	2021/4/6
WKN88562	P.1	38	ORF10 protein	2021/4/6
WKN88690	B.1.621	7094	ORF1ab polyprotein	2021/5/27
WKN88691	B.1.621	4403	ORF1a polyprotein	2021/5/27

WKN88692	B.1.621	1274	surface glycoprotein	2021/5/27
WKN88693	B.1.621	75	envelope protein	2021/5/27
WKN88694	B.1.621	222	membrane glycoprotein	2021/5/27
WKN88695	B.1.621	61	ORF6 protein	2021/5/27
WKN88696	B.1.621	121	ORF7a protein	2021/5/27
WKN88697	B.1.621	43	ORF7b protein	2021/5/27
WKN88698	B.1.621	121	ORF8 protein	2021/5/27
WKN88699	B.1.621	419	nucleocapsid phosphoprotein	2021/5/27
WKN88700	B.1.621	38	ORF10 protein	2021/5/27
WKN94439	B.1.351	7093	ORF1ab polyprotein	2021/4/1
WKN94440	B.1.351	4402	ORF1a polyprotein	2021/4/1
WKN94441	B.1.351	1270	surface glycoprotein	2021/4/1
WKN94442	B.1.351	275	ORF3a protein	2021/4/1
WKN94443	B.1.351	75	envelope protein	2021/4/1
WKN94444	B.1.351	222	membrane glycoprotein	2021/4/1
WKN94445	B.1.351	61	ORF6 protein	2021/4/1
WKN94446	B.1.351	121	ORF7a protein	2021/4/1
WKN94447	B.1.351	43	ORF7b protein	2021/4/1
WKN94448	B.1.351	121	ORF8 protein	2021/4/1
WKN94449	B.1.351	419	nucleocapsid phosphoprotein	2021/4/1
WKN94450	B.1.351	38	ORF10 protein	2021/4/1
WKO21592	P.1.15	7093	ORF1ab polyprotein	2021/4/17
WKO21593	P.1.15	4402	ORF1a polyprotein	2021/4/17
WKO21594	P.1.15	1273	surface glycoprotein	2021/4/17
WKO21595	P.1.15	275	ORF3a protein	2021/4/17
WKO21596	P.1.15	75	envelope protein	2021/4/17
WKO21597	P.1.15	222	membrane glycoprotein	2021/4/17
WKO21598	P.1.15	61	ORF6 protein	2021/4/17
WKO21599	P.1.15	121	ORF7a protein	2021/4/17
WKO21600	P.1.15	43	ORF7b protein	2021/4/17
WKO21601	P.1.15	121	ORF8 protein	2021/4/17
WKO21602	P.1.15	419	nucleocapsid phosphoprotein	2021/4/17
WKO21603	P.1.15	38	ORF10 protein	2021/4/17
WKO25912	BA.1.15	7092	ORF1ab polyprotein	2021/12/31
WKO25913	BA.1.15	4401	ORF1a polyprotein	2021/12/31
WKO25914	BA.1.15	1270	surface glycoprotein	2021/12/31
WKO25915	BA.1.15	275	ORF3a protein	2021/12/31
WKO25916	BA.1.15	75	envelope protein	2021/12/31
WKO25917	BA.1.15	222	membrane glycoprotein	2021/12/31
WKO25918	BA.1.15	61	ORF6 protein	2021/12/31
WKO25919	BA.1.15	121	ORF7a protein	2021/12/31
WKO25920	BA.1.15	43	ORF7b protein	2021/12/31

WKO25921	BA.1.15	121	ORF8 protein	2021/12/31
WKO25922	BA.1.15	416	nucleocapsid phosphoprotein	2021/12/31
WKO25923	BA.1.15	38	ORF10 protein	2021/12/31
WKO30551	AY.43	7096	ORF1ab polyprotein	2021/8/18
WKO30552	AY.43	4405	ORF1a polyprotein	2021/8/18
WKO30553	AY.43	1271	surface glycoprotein	2021/8/18
WKO30554	AY.43	275	ORF3a protein	2021/8/18
WKO30555	AY.43	75	envelope protein	2021/8/18
WKO30556	AY.43	222	membrane glycoprotein	2021/8/18
WKO30557	AY.43	61	ORF6 protein	2021/8/18
WKO30558	AY.43	121	ORF7a protein	2021/8/18
WKO30559	AY.43	43	ORF7b protein	2021/8/18
WKO30560	AY.43	119	ORF8 protein	2021/8/18
WKO30561	AY.43	419	nucleocapsid phosphoprotein	2021/8/18
WKO30562	AY.43	38	ORF10 protein	2021/8/18
WKO43786	BA.1.1	7092	ORF1ab polyprotein	2022/2/8
WKO43787	BA.1.1	4401	ORF1a polyprotein	2022/2/8
WKO43788	BA.1.1	1270	surface glycoprotein	2022/2/8
WKO43789	BA.1.1	275	ORF3a protein	2022/2/8
WKO43790	BA.1.1	75	envelope protein	2022/2/8
WKO43791	BA.1.1	222	membrane glycoprotein	2022/2/8
WKO43792	BA.1.1	61	ORF6 protein	2022/2/8
WKO43793	BA.1.1	121	ORF7a protein	2022/2/8
WKO43794	BA.1.1	43	ORF7b protein	2022/2/8
WKO43795	BA.1.1	121	ORF8 protein	2022/2/8
WKO43796	BA.1.1	416	nucleocapsid phosphoprotein	2022/2/8
WKO43797	BA.1.1	38	ORF10 protein	2022/2/8
WKO65537	B.1.525	7093	ORF1ab polyprotein	2021/2/26
WKO65538	B.1.525	4402	ORF1a polyprotein	2021/2/26
WKO65539	B.1.525	1270	surface glycoprotein	2021/2/26
WKO65540	B.1.525	275	ORF3a protein	2021/2/26
WKO65541	B.1.525	75	envelope protein	2021/2/26
WKO65542	B.1.525	222	membrane glycoprotein	2021/2/26
WKO65543	B.1.525	60	ORF6 protein	2021/2/26
WKO65544	B.1.525	121	ORF7a protein	2021/2/26
WKO65545	B.1.525	43	ORF7b protein	2021/2/26
WKO65546	B.1.525	121	ORF8 protein	2021/2/26
WKO65547	B.1.525	418	nucleocapsid phosphoprotein	2021/2/26
WKO65548	B.1.525	38	ORF10 protein	2021/2/26
WKO69653	BA.1	7092	ORF1ab polyprotein	2021/12/25
WKO69654	BA.1	4401	ORF1a polyprotein	2021/12/25
WKO69655	BA.1	1270	surface glycoprotein	2021/12/25

WKO69656	BA.1	275	ORF3a protein	2021/12/25
WKO69657	BA.1	75	envelope protein	2021/12/25
WKO69658	BA.1	222	membrane glycoprotein	2021/12/25
WKO69659	BA.1	61	ORF6 protein	2021/12/25
WKO69660	BA.1	121	ORF7a protein	2021/12/25
WKO69661	BA.1	29	ORF7b protein	2021/12/25
WKO69662	BA.1	56	ORF8 protein	2021/12/25
WKO69663	BA.1	416	nucleocapsid phosphoprotein	2021/12/25
WKO69664	BA.1	38	ORF10 protein	2021/12/25
WKO98994	AY.4	7096	ORF1ab polyprotein	2021/11/3
WKO98995	AY.4	4405	ORF1a polyprotein	2021/11/3
WKO98996	AY.4	1271	surface glycoprotein	2021/11/3
WKO98997	AY.4	275	ORF3a protein	2021/11/3
WKO98998	AY.4	75	envelope protein	2021/11/3
WKO98999	AY.4	222	membrane glycoprotein	2021/11/3
WKO99000	AY.4	61	ORF6 protein	2021/11/3
WKO99001	AY.4	121	ORF7a protein	2021/11/3
WKO99002	AY.4	43	ORF7b protein	2021/11/3
WKO99003	AY.4	119	ORF8 protein	2021/11/3
WKO99004	AY.4	419	nucleocapsid phosphoprotein	2021/11/3
WKO99005	AY.4	38	ORF10 protein	2021/11/3
WKP42178	P.1.17	7093	ORF1ab polyprotein	2021/5/13
WKP42179	P.1.17	4402	ORF1a polyprotein	2021/5/13
WKP42180	P.1.17	1273	surface glycoprotein	2021/5/13
WKP42181	P.1.17	275	ORF3a protein	2021/5/13
WKP42182	P.1.17	75	envelope protein	2021/5/13
WKP42183	P.1.17	222	membrane glycoprotein	2021/5/13
WKP42184	P.1.17	61	ORF6 protein	2021/5/13
WKP42185	P.1.17	121	ORF7a protein	2021/5/13
WKP42186	P.1.17	43	ORF7b protein	2021/5/13
WKP42187	P.1.17	121	ORF8 protein	2021/5/13
WKP42188	P.1.17	419	nucleocapsid phosphoprotein	2021/5/13
WKP42189	P.1.17	38	ORF10 protein	2021/5/13
WKP68573	B.1.621.1	7096	ORF1ab polyprotein	2021/7/4
WKP68574	B.1.621.1	4405	ORF1a polyprotein	2021/7/4
WKP68575	B.1.621.1	1274	surface glycoprotein	2021/7/4
WKP68576	B.1.621.1	75	envelope protein	2021/7/4
WKP68577	B.1.621.1	222	membrane glycoprotein	2021/7/4
WKP68578	B.1.621.1	61	ORF6 protein	2021/7/4
WKP68579	B.1.621.1	121	ORF7a protein	2021/7/4
WKP68580	B.1.621.1	43	ORF7b protein	2021/7/4
WKP68581	B.1.621.1	121	ORF8 protein	2021/7/4

WKP68582	B.1.621.1	419	nucleocapsid phosphoprotein	2021/7/4
WKP68583	B.1.621.1	38	ORF10 protein	2021/7/4
WKQ50333	BA.1.17.2	7092	ORF1ab polyprotein	2022/1/3
WKQ50334	BA.1.17.2	4401	ORF1a polyprotein	2022/1/3
WKQ50335	BA.1.17.2	1270	surface glycoprotein	2022/1/3
WKQ50336	BA.1.17.2	275	ORF3a protein	2022/1/3
WKQ50337	BA.1.17.2	75	envelope protein	2022/1/3
WKQ50338	BA.1.17.2	222	membrane glycoprotein	2022/1/3
WKQ50339	BA.1.17.2	61	ORF6 protein	2022/1/3
WKQ50340	BA.1.17.2	121	ORF7a protein	2022/1/3
WKQ50341	BA.1.17.2	43	ORF7b protein	2022/1/3
WKQ50342	BA.1.17.2	121	ORF8 protein	2022/1/3
WKQ50343	BA.1.17.2	416	nucleocapsid phosphoprotein	2022/1/3
WKQ50344	BA.1.17.2	38	ORF10 protein	2022/1/3
WKQ68073	B.1.351.2	7070	ORF1ab polyprotein	Jan-21
WKQ68074	B.1.351.2	4379	ORF1a polyprotein	Jan-21
WKQ68075	B.1.351.2	1270	surface glycoprotein	Jan-21
WKQ68076	B.1.351.2	275	ORF3a protein	Jan-21
WKQ68077	B.1.351.2	75	envelope protein	Jan-21
WKQ68078	B.1.351.2	222	membrane glycoprotein	Jan-21
WKQ68079	B.1.351.2	61	ORF6 protein	Jan-21
WKQ68080	B.1.351.2	121	ORF7a protein	Jan-21
WKQ68081	B.1.351.2	43	ORF7b protein	Jan-21
WKQ68082	B.1.351.2	121	ORF8 protein	Jan-21
WKQ68083	B.1.351.2	419	nucleocapsid phosphoprotein	Jan-21
WKQ68084	B.1.351.2	38	ORF10 protein	Jan-21
WKQ70717	P.1.7	7093	ORF1ab polyprotein	Apr-21
WKQ70718	P.1.7	4402	ORF1a polyprotein	Apr-21
WKQ70719	P.1.7	1270	surface glycoprotein	Apr-21
WKQ70720	P.1.7	275	ORF3a protein	Apr-21
WKQ70721	P.1.7	75	envelope protein	Apr-21
WKQ70722	P.1.7	222	membrane glycoprotein	Apr-21
WKQ70723	P.1.7	61	ORF6 protein	Apr-21
WKQ70724	P.1.7	121	ORF7a protein	Apr-21
WKQ70725	P.1.7	43	ORF7b protein	Apr-21
WKQ70726	P.1.7	419	nucleocapsid phosphoprotein	Apr-21
WKQ70727	P.1.7	38	ORF10 protein	Apr-21
WKQ78309	AY.4.2	7096	ORF1ab polyprotein	Nov-21
WKQ78310	AY.4.2	4405	ORF1a polyprotein	Nov-21
WKQ78311	AY.4.2	1271	surface glycoprotein	Nov-21
WKQ78312	AY.4.2	275	ORF3a protein	Nov-21
WKQ78313	AY.4.2	75	envelope protein	Nov-21

WKQ78314	AY.4.2	222	membrane glycoprotein	Nov-21
WKQ78315	AY.4.2	61	ORF6 protein	Nov-21
WKQ78316	AY.4.2	121	ORF7a protein	Nov-21
WKQ78317	AY.4.2	43	ORF7b protein	Nov-21
WKQ78318	AY.4.2	119	ORF8 protein	Nov-21
WKQ78319	AY.4.2	419	nucleocapsid phosphoprotein	Nov-21
WKQ78320	AY.4.2	38	ORF10 protein	Nov-21
WKR17129	BQ.1	7087	ORF1ab polyprotein	Sep-22
WKR17130	BQ.1	4396	ORF1a polyprotein	Sep-22
WKR17131	BQ.1	1268	surface glycoprotein	Sep-22
WKR17132	BQ.1	275	ORF3a protein	Sep-22
WKR17133	BQ.1	75	envelope protein	Sep-22
WKR17134	BQ.1	222	membrane glycoprotein	Sep-22
WKR17135	BQ.1	61	ORF6 protein	Sep-22
WKR17136	BQ.1	121	ORF7a protein	Sep-22
WKR17137	BQ.1	121	ORF8 protein	Sep-22
WKR17138	BQ.1	416	nucleocapsid phosphoprotein	Sep-22
WKR17139	BQ.1	38	ORF10 protein	Sep-22
WKR17152	BQ.1.1.4	7093	ORF1ab polyprotein	Oct-22
WKR17153	BQ.1.1.4	4402	ORF1a polyprotein	Oct-22
WKR17154	BQ.1.1.4	1268	surface glycoprotein	Oct-22
WKR17155	BQ.1.1.4	275	ORF3a protein	Oct-22
WKR17156	BQ.1.1.4	75	envelope protein	Oct-22
WKR17157	BQ.1.1.4	222	membrane glycoprotein	Oct-22
WKR17158	BQ.1.1.4	61	ORF6 protein	Oct-22
WKR17159	BQ.1.1.4	121	ORF7a protein	Oct-22
WKR17160	BQ.1.1.4	43	ORF7b protein	Oct-22
WKR17161	BQ.1.1.4	121	ORF8 protein	Oct-22
WKR17162	BQ.1.1.4	416	nucleocapsid phosphoprotein	Oct-22
WKR17163	BQ.1.1.4	38	ORF10 protein	Oct-22
WKR17260	BQ.1.1.3	7093	ORF1ab polyprotein	Oct-22
WKR17261	BQ.1.1.3	4402	ORF1a polyprotein	Oct-22
WKR17262	BQ.1.1.3	1268	surface glycoprotein	Oct-22
WKR17263	BQ.1.1.3	275	ORF3a protein	Oct-22
WKR17264	BQ.1.1.3	75	envelope protein	Oct-22
WKR17265	BQ.1.1.3	222	membrane glycoprotein	Oct-22
WKR17266	BQ.1.1.3	61	ORF6 protein	Oct-22
WKR17267	BQ.1.1.3	121	ORF7a protein	Oct-22
WKR17268	BQ.1.1.3	43	ORF7b protein	Oct-22
WKR17269	BQ.1.1.3	121	ORF8 protein	Oct-22
WKR17270	BQ.1.1.3	416	nucleocapsid phosphoprotein	Oct-22
WKR17271	BQ.1.1.3	38	ORF10 protein	Oct-22

WKL12723	BQ.1.1.18	7067	ORF1ab polyprotein	Oct-22
WKL12724	BQ.1.1.18	4337	ORF1a polyprotein	Oct-22
WKL12725	BQ.1.1.18	1268	surface glycoprotein	Oct-22
WKL12726	BQ.1.1.18	275	ORF3a protein	Oct-22
WKL12727	BQ.1.1.18	75	envelope protein	Oct-22
WKL12728	BQ.1.1.18	222	membrane glycoprotein	Oct-22
WKL12729	BQ.1.1.18	61	ORF6 protein	Oct-22
WKL12730	BQ.1.1.18	39	ORF7a protein	Oct-22
WKL12731	BQ.1.1.18	111	ORF8 protein	Oct-22
WKL12732	BQ.1.1.18	416	nucleocapsid phosphoprotein	Oct-22
WKL12733	BQ.1.1.18	31	ORF10 protein	Oct-22
WKL13117	BQ.1.8	7041	ORF1ab polyprotein	Nov-22
WKL13118	BQ.1.8	4350	ORF1a polyprotein	Nov-22
WKL13119	BQ.1.8	1267	surface glycoprotein	Nov-22
WKL13120	BQ.1.8	275	ORF3a protein	Nov-22
WKL13121	BQ.1.8	75	envelope protein	Nov-22
WKL13122	BQ.1.8	222	membrane glycoprotein	Nov-22
WKL13123	BQ.1.8	61	ORF6 protein	Nov-22
WKL13124	BQ.1.8	121	ORF7a protein	Nov-22
WKL13125	BQ.1.8	6	ORF7b protein	Nov-22
WKL13126	BQ.1.8	50	ORF8 protein	Nov-22
WKL13127	BQ.1.8	416	nucleocapsid phosphoprotein	Nov-22
WKL13128	BQ.1.8	36	ORF10 protein	Nov-22
WKL13254	BQ.1.2	7067	ORF1ab polyprotein	Nov-22
WKL13255	BQ.1.2	4376	ORF1a polyprotein	Nov-22
WKL13256	BQ.1.2	1268	surface glycoprotein	Nov-22
WKL13257	BQ.1.2	275	ORF3a protein	Nov-22
WKL13258	BQ.1.2	75	envelope protein	Nov-22
WKL13259	BQ.1.2	222	membrane glycoprotein	Nov-22
WKL13260	BQ.1.2	61	ORF6 protein	Nov-22
WKL13261	BQ.1.2	121	ORF7a protein	Nov-22
WKL13262	BQ.1.2	43	ORF7b protein	Nov-22
WKL13263	BQ.1.2	121	ORF8 protein	Nov-22
WKL13264	BQ.1.2	416	nucleocapsid phosphoprotein	Nov-22
WKL13265	BQ.1.2	38	ORF10 protein	Nov-22
WKL05900	XBB.2.3.10	7093	ORF1ab polyprotein	2023/6/11
WKL05901	XBB.2.3.10	4402	ORF1a polyprotein	2023/6/11
WKL05902	XBB.2.3.10	1269	surface glycoprotein	2023/6/11
WKL05903	XBB.2.3.10	275	ORF3a protein	2023/6/11
WKL05904	XBB.2.3.10	75	envelope protein	2023/6/11
WKL05905	XBB.2.3.10	222	membrane glycoprotein	2023/6/11
WKL05906	XBB.2.3.10	61	ORF6 protein	2023/6/11

WKL05907	XBB.2.3.10	121	ORF7a protein	2023/6/11
WKL05908	XBB.2.3.10	43	ORF7b protein	2023/6/11
WKL05909	XBB.2.3.10	121	ORF8 protein	2023/6/11
WKL05910	XBB.2.3.10	416	nucleocapsid phosphoprotein	2023/6/11
WKL05911	XBB.2.3.10	38	ORF10 protein	2023/6/11
WKI74658	BA.2.75.2	7066	ORF1ab polyprotein	Sep-22
WKI74659	BA.2.75.2	4375	ORF1a polyprotein	Sep-22
WKI74660	BA.2.75.2	1270	surface glycoprotein	Sep-22
WKI74661	BA.2.75.2	275	ORF3a protein	Sep-22
WKI74662	BA.2.75.2	75	envelope protein	Sep-22
WKI74663	BA.2.75.2	222	membrane glycoprotein	Sep-22
WKI74664	BA.2.75.2	61	ORF6 protein	Sep-22
WKI74665	BA.2.75.2	121	ORF7a protein	Sep-22
WKI74666	BA.2.75.2	43	ORF7b protein	Sep-22
WKI74667	BA.2.75.2	121	ORF8 protein	Sep-22
WKI74668	BA.2.75.2	416	nucleocapsid phosphoprotein	Sep-22
WKI74669	BA.2.75.2	38	ORF10 protein	Sep-22
WKI76157	BA.2.75.1	7067	ORF1ab polyprotein	Sep-22
WKI76158	BA.2.75.1	4376	ORF1a polyprotein	Sep-22
WKI76159	BA.2.75.1	1270	surface glycoprotein	Sep-22
WKI76160	BA.2.75.1	275	ORF3a protein	Sep-22
WKI76161	BA.2.75.1	75	envelope protein	Sep-22
WKI76162	BA.2.75.1	222	membrane glycoprotein	Sep-22
WKI76163	BA.2.75.1	61	ORF6 protein	Sep-22
WKI76164	BA.2.75.1	121	ORF7a protein	Sep-22
WKI76165	BA.2.75.1	43	ORF7b protein	Sep-22
WKI76166	BA.2.75.1	121	ORF8 protein	Sep-22
WKI76167	BA.2.75.1	416	nucleocapsid phosphoprotein	Sep-22
WKI76168	BA.2.75.1	38	ORF10 protein	Sep-22
WKK31597	XBB.1.5.20	4399	ORF1a polyprotein	2023/5/13
WKK31598	XBB.1.5.20	7077	ORF1ab polyprotein	2023/5/13
WKK31599	XBB.1.5.20	1257	surface glycoprotein	2023/5/13
WKK31600	XBB.1.5.20	275	ORF3a protein	2023/5/13
WKK31601	XBB.1.5.20	75	envelope protein	2023/5/13
WKK31602	XBB.1.5.20	222	membrane glycoprotein	2023/5/13
WKK31603	XBB.1.5.20	42	ORF6 protein	2023/5/13
WKK31604	XBB.1.5.20	121	ORF7a protein	2023/5/13
WKK31605	XBB.1.5.20	43	ORF7b protein	2023/5/13
WKK31606	XBB.1.5.20	416	nucleocapsid phosphoprotein	2023/5/13
WKK31607	XBB.1.5.20	38	ORF10 protein	2023/5/13
WKK31674	CH.1.1.1	4402	ORF1a polyprotein	2023/5/16
WKK31675	CH.1.1.1	7080	ORF1ab polyprotein	2023/5/16

WKK31676	CH.1.1.1	1258	surface glycoprotein	2023/5/16
WKK31677	CH.1.1.1	275	ORF3a protein	2023/5/16
WKK31678	CH.1.1.1	75	envelope protein	2023/5/16
WKK31679	CH.1.1.1	222	membrane glycoprotein	2023/5/16
WKK31680	CH.1.1.1	6	ORF6 protein	2023/5/16
WKK31681	CH.1.1.1	120	ORF7a protein	2023/5/16
WKK31682	CH.1.1.1	43	ORF7b protein	2023/5/16
WKK31683	CH.1.1.1	416	nucleocapsid phosphoprotein	2023/5/16
WKK31684	CH.1.1.1	38	ORF10 protein	2023/5/16
WKK50887	CH.1.1	7067	ORF1ab polyprotein	2023/2/15
WKK50888	CH.1.1	4376	ORF1a polyprotein	2023/2/15
WKK50889	CH.1.1	1270	surface glycoprotein	2023/2/15
WKK50890	CH.1.1	275	ORF3a protein	2023/2/15
WKK50891	CH.1.1	75	envelope protein	2023/2/15
WKK50892	CH.1.1	222	membrane glycoprotein	2023/2/15
WKK50893	CH.1.1	61	ORF6 protein	2023/2/15
WKK50894	CH.1.1	121	ORF7a protein	2023/2/15
WKK50895	CH.1.1	43	ORF7b protein	2023/2/15
WKK50896	CH.1.1	121	ORF8 protein	2023/2/15
WKK50897	CH.1.1	416	nucleocapsid phosphoprotein	2023/2/15
WKK50898	CH.1.1	38	ORF10 protein	2023/2/15
WKF16625	B.1.1.529	7092	ORF1ab polyprotein	2021/12/8
WKF16626	B.1.1.529	4401	ORF1a polyprotein	2021/12/8
WKF16627	B.1.1.529	1270	surface glycoprotein	2021/12/8
WKF16628	B.1.1.529	275	ORF3a protein	2021/12/8
WKF16629	B.1.1.529	75	envelope protein	2021/12/8
WKF16630	B.1.1.529	222	membrane glycoprotein	2021/12/8
WKF16631	B.1.1.529	61	ORF6 protein	2021/12/8
WKF16632	B.1.1.529	43	ORF7a protein	2021/12/8
WKF16633	B.1.1.529	58	ORF8 protein	2021/12/8
WKF16634	B.1.1.529	416	nucleocapsid phosphoprotein	2021/12/8
WKF16635	B.1.1.529	38	ORF10 protein	2021/12/8
WKB16851	BQ.1.12	7093	ORF1ab polyprotein	2023/1/6
WKB16852	BQ.1.12	4402	ORF1a polyprotein	2023/1/6
WKB16853	BQ.1.12	1268	surface glycoprotein	2023/1/6
WKB16854	BQ.1.12	275	ORF3a protein	2023/1/6
WKB16855	BQ.1.12	75	envelope protein	2023/1/6
WKB16856	BQ.1.12	222	membrane glycoprotein	2023/1/6
WKB16857	BQ.1.12	61	ORF6 protein	2023/1/6
WKB16858	BQ.1.12	121	ORF7a protein	2023/1/6
WKB16859	BQ.1.12	43	ORF7b protein	2023/1/6
WKB16860	BQ.1.12	121	ORF8 protein	2023/1/6

WKB16861	BQ.1.12	416	nucleocapsid phosphoprotein	2023/1/6
WKB16862	BQ.1.12	38	ORF10 protein	2023/1/6
WJP35161	BQ.1.14	4402	ORF1a polyprotein	2022/11/20
WJP35162	BQ.1.14	7080	ORF1ab polyprotein	2022/11/20
WJP35163	BQ.1.14	1225	surface glycoprotein	2022/11/20
WJP35164	BQ.1.14	275	ORF3a protein	2022/11/20
WJP35165	BQ.1.14	68	envelope protein	2022/11/20
WJP35166	BQ.1.14	208	membrane glycoprotein	2022/11/20
WJP35167	BQ.1.14	60	ORF6 protein	2022/11/20
WJP35168	BQ.1.14	121	ORF7a protein	2022/11/20
WJP35169	BQ.1.14	23	ORF7b protein	2022/11/20
WJP35170	BQ.1.14	61	ORF8 protein	2022/11/20
WJP35171	BQ.1.14	394	nucleocapsid phosphoprotein	2022/11/20
WJP35172	BQ.1.14	36	ORF10 protein	2022/11/20
WJP38839	BA.2.75.5	7093	ORF1ab polyprotein	2022/11/28
WJP38840	BA.2.75.5	4402	ORF1a polyprotein	2022/11/28
WJP38841	BA.2.75.5	1270	surface glycoprotein	2022/11/28
WJP38842	BA.2.75.5	275	ORF3a protein	2022/11/28
WJP38843	BA.2.75.5	75	envelope protein	2022/11/28
WJP38844	BA.2.75.5	222	membrane glycoprotein	2022/11/28
WJP38845	BA.2.75.5	61	ORF6 protein	2022/11/28
WJP38846	BA.2.75.5	121	ORF7a protein	2022/11/28
WJP38847	BA.2.75.5	43	ORF7b protein	2022/11/28
WJP38848	BA.2.75.5	121	ORF8 protein	2022/11/28
WJP38849	BA.2.75.5	416	nucleocapsid phosphoprotein	2022/11/28
WJP38850	BA.2.75.5	38	ORF10 protein	2022/11/28
WJP41215	CH.1.1.25	4402	ORF1a polyprotein	2022/12/7
WJP41216	CH.1.1.25	7080	ORF1ab polyprotein	2022/12/7
WJP41217	CH.1.1.25	1261	surface glycoprotein	2022/12/7
WJP41218	CH.1.1.25	275	ORF3a protein	2022/12/7
WJP41219	CH.1.1.25	75	envelope protein	2022/12/7
WJP41220	CH.1.1.25	190	membrane glycoprotein	2022/12/7
WJP41221	CH.1.1.25	61	ORF6 protein	2022/12/7
WJP41222	CH.1.1.25	121	ORF7a protein	2022/12/7
WJP41223	CH.1.1.25	43	ORF7b protein	2022/12/7
WJP41224	CH.1.1.25	121	ORF8 protein	2022/12/7
WJP41225	CH.1.1.25	416	nucleocapsid phosphoprotein	2022/12/7
WJP41226	CH.1.1.25	36	ORF10 protein	2022/12/7
WJP54157	BA.2.75	7093	ORF1ab polyprotein	2022/11/21
WJP54158	BA.2.75	4402	ORF1a polyprotein	2022/11/21
WJP54159	BA.2.75	1270	surface glycoprotein	2022/11/21
WJP54160	BA.2.75	275	ORF3a protein	2022/11/21

WJP54161	BA.2.75	75	envelope protein	2022/11/21
WJP54162	BA.2.75	222	membrane glycoprotein	2022/11/21
WJP54163	BA.2.75	61	ORF6 protein	2022/11/21
WJP54164	BA.2.75	121	ORF7a protein	2022/11/21
WJP54165	BA.2.75	43	ORF7b protein	2022/11/21
WJP54166	BA.2.75	121	ORF8 protein	2022/11/21
WJP54167	BA.2.75	416	nucleocapsid phosphoprotein	2022/11/21
WJP54168	BA.2.75	38	ORF10 protein	2022/11/21
WJR92519	C.37	7093	ORF1ab polyprotein	2021/8/1
WJR92520	C.37	4382	ORF1a polyprotein	2021/8/1
WJR92521	C.37	1273	surface glycoprotein	2021/8/1
WJR92522	C.37	275	ORF3a protein	2021/8/1
WJR92523	C.37	75	envelope protein	2021/8/1
WJR92524	C.37	222	membrane glycoprotein	2021/8/1
WJR92525	C.37	61	ORF6 protein	2021/8/1
WJR92526	C.37	44	ORF7a protein	2021/8/1
WJR92527	C.37	30	ORF7b protein	2021/8/1
WJR92528	C.37	121	ORF8 protein	2021/8/1
WJR92529	C.37	419	nucleocapsid phosphoprotein	2021/8/1
WJR92530	C.37	38	ORF10 protein	2021/8/1
WJN42231	CH.1.1.11	7090	ORF1ab polyprotein	2023/5/10
WJN42232	CH.1.1.11	4399	ORF1a polyprotein	2023/5/10
WJN42233	CH.1.1.11	1269	surface glycoprotein	2023/5/10
WJN42234	CH.1.1.11	275	ORF3a protein	2023/5/10
WJN42235	CH.1.1.11	75	envelope protein	2023/5/10
WJN42236	CH.1.1.11	222	membrane glycoprotein	2023/5/10
WJN42237	CH.1.1.11	61	ORF6 protein	2023/5/10
WJN42238	CH.1.1.11	121	ORF7a protein	2023/5/10
WJN42239	CH.1.1.11	43	ORF7b protein	2023/5/10
WJN42240	CH.1.1.11	416	nucleocapsid phosphoprotein	2023/5/10
WJN42241	CH.1.1.11	38	ORF10 protein	2023/5/10
WJN02979	CH.1.1.10	4209	ORF1a polyprotein	2023/3/1
WJN02980	CH.1.1.10	6608	ORF1ab polyprotein	2023/3/1
WJN02981	CH.1.1.10	948	surface glycoprotein	2023/3/1
WJN02982	CH.1.1.10	275	ORF3a protein	2023/3/1
WJN02983	CH.1.1.10	75	envelope protein	2023/3/1
WJN02984	CH.1.1.10	222	membrane glycoprotein	2023/3/1
WJN02985	CH.1.1.10	61	ORF6 protein	2023/3/1
WJN02986	CH.1.1.10	121	ORF7a protein	2023/3/1
WJN02987	CH.1.1.10	43	ORF7b protein	2023/3/1
WJN02988	CH.1.1.10	121	ORF8 protein	2023/3/1
WJN02989	CH.1.1.10	416	nucleocapsid phosphoprotein	2023/3/1

WJN02990	CH.1.1.10	38	ORF10 protein	2023/3/1
WIJ13456	BA.2.75.4	7093	ORF1ab polyprotein	2022/8/8
WIJ13457	BA.2.75.4	4402	ORF1a polyprotein	2022/8/8
WIJ13458	BA.2.75.4	1270	surface glycoprotein	2022/8/8
WIJ13459	BA.2.75.4	275	ORF3a protein	2022/8/8
WIJ13460	BA.2.75.4	75	envelope protein	2022/8/8
WIJ13461	BA.2.75.4	222	membrane glycoprotein	2022/8/8
WIJ13462	BA.2.75.4	61	ORF6 protein	2022/8/8
WIJ13463	BA.2.75.4	121	ORF7a protein	2022/8/8
WIJ13464	BA.2.75.4	43	ORF7b protein	2022/8/8
WIJ13465	BA.2.75.4	121	ORF8 protein	2022/8/8
WIJ13466	BA.2.75.4	416	nucleocapsid phosphoprotein	2022/8/8
WIJ13467	BA.2.75.4	38	ORF10 protein	2022/8/8
WIG64434	Q.3	7093	ORF1ab polyprotein	2021/4/19
WIG64435	Q.3	4402	ORF1a polyprotein	2021/4/19
WIG64436	Q.3	1270	surface glycoprotein	2021/4/19
WIG64437	Q.3	275	ORF3a protein	2021/4/19
WIG64438	Q.3	75	envelope protein	2021/4/19
WIG64439	Q.3	222	membrane glycoprotein	2021/4/19
WIG64440	Q.3	61	ORF6 protein	2021/4/19
WIG64441	Q.3	121	ORF7a protein	2021/4/19
WIG64442	Q.3	43	ORF7b protein	2021/4/19
WIG64443	Q.3	419	nucleocapsid phosphoprotein	2021/4/19
WIG64444	Q.3	38	ORF10 protein	2021/4/19
WHR80278	CH.1.1.13	7093	ORF1ab polyprotein	2023/1/3
WHR80279	CH.1.1.13	4402	ORF1a polyprotein	2023/1/3
WHR80280	CH.1.1.13	1270	surface glycoprotein	2023/1/3
WHR80281	CH.1.1.13	275	ORF3a protein	2023/1/3
WHR80282	CH.1.1.13	75	envelope protein	2023/1/3
WHR80283	CH.1.1.13	222	membrane glycoprotein	2023/1/3
WHR80284	CH.1.1.13	61	ORF6 protein	2023/1/3
WHR80285	CH.1.1.13	121	ORF7a protein	2023/1/3
WHR80286	CH.1.1.13	43	ORF7b protein	2023/1/3
WHR80287	CH.1.1.13	121	ORF8 protein	2023/1/3
WHR80288	CH.1.1.13	416	nucleocapsid phosphoprotein	2023/1/3
WHR80289	CH.1.1.13	38	ORF10 protein	2023/1/3
WGU65652	XBB.1.16.7	7093	ORF1ab polyprotein	2023/4/17
WGU65653	XBB.1.16.7	4402	ORF1a polyprotein	2023/4/17
WGU65654	XBB.1.16.7	1269	surface glycoprotein	2023/4/17
WGU65655	XBB.1.16.7	275	ORF3a protein	2023/4/17
WGU65656	XBB.1.16.7	75	envelope protein	2023/4/17
WGU65657	XBB.1.16.7	222	membrane glycoprotein	2023/4/17

WGU65658	XBB.1.16.7	61	ORF6 protein	2023/4/17
WGU65659	XBB.1.16.7	121	ORF7a protein	2023/4/17
WGU65660	XBB.1.16.7	43	ORF7b protein	2023/4/17
WGU65661	XBB.1.16.7	416	nucleocapsid phosphoprotein	2023/4/17
WGU65662	XBB.1.16.7	38	ORF10 protein	2023/4/17
WGQ46284	BA.2.75.3	7088	ORF1ab polyprotein	Aug-22
WGQ46285	BA.2.75.3	4397	ORF1a polyprotein	Aug-22
WGQ46286	BA.2.75.3	1270	surface glycoprotein	Aug-22
WGQ46287	BA.2.75.3	275	ORF3a protein	Aug-22
WGQ46288	BA.2.75.3	75	envelope protein	Aug-22
WGQ46289	BA.2.75.3	222	membrane glycoprotein	Aug-22
WGQ46290	BA.2.75.3	61	ORF6 protein	Aug-22
WGQ46291	BA.2.75.3	121	ORF7a protein	Aug-22
WGQ46292	BA.2.75.3	43	ORF7b protein	Aug-22
WGQ46293	BA.2.75.3	121	ORF8 protein	Aug-22
WGQ46294	BA.2.75.3	416	nucleocapsid phosphoprotein	Aug-22
WGQ46295	BA.2.75.3	38	ORF10 protein	Aug-22
WGQ46848	BA.2.75.10	7093	ORF1ab polyprotein	Sep-22
WGQ46849	BA.2.75.10	4402	ORF1a polyprotein	Sep-22
WGQ46850	BA.2.75.10	1270	surface glycoprotein	Sep-22
WGQ46851	BA.2.75.10	275	ORF3a protein	Sep-22
WGQ46852	BA.2.75.10	75	envelope protein	Sep-22
WGQ46853	BA.2.75.10	222	membrane glycoprotein	Sep-22
WGQ46854	BA.2.75.10	61	ORF6 protein	Sep-22
WGQ46855	BA.2.75.10	121	ORF7a protein	Sep-22
WGQ46856	BA.2.75.10	43	ORF7b protein	Sep-22
WGQ46857	BA.2.75.10	121	ORF8 protein	Sep-22
WGQ46858	BA.2.75.10	416	nucleocapsid phosphoprotein	Sep-22
WGQ46859	BA.2.75.10	38	ORF10 protein	Sep-22
WGQ89765	P.1.1	7070	ORF1ab polyprotein	May-21
WGQ89766	P.1.1	4343	ORF1a polyprotein	May-21
WGQ89767	P.1.1	1273	surface glycoprotein	May-21
WGQ89768	P.1.1	275	ORF3a protein	May-21
WGQ89769	P.1.1	75	envelope protein	May-21
WGQ89770	P.1.1	222	membrane glycoprotein	May-21
WGQ89771	P.1.1	61	ORF6 protein	May-21
WGQ89772	P.1.1	121	ORF7a protein	May-21
WGQ89773	P.1.1	43	ORF7b protein	May-21
WGQ89774	P.1.1	121	ORF8 protein	May-21
WGQ89775	P.1.1	419	nucleocapsid phosphoprotein	May-21
WGQ89776	P.1.1	38	ORF10 protein	May-21
WGJ80001	BA.2.75.6	7093	ORF1ab polyprotein	2023/4/10

WGJ80002	BA.2.75.6	4402	ORF1a polyprotein	2023/4/10
WGJ80003	BA.2.75.6	1270	surface glycoprotein	2023/4/10
WGJ80004	BA.2.75.6	275	ORF3a protein	2023/4/10
WGJ80005	BA.2.75.6	75	envelope protein	2023/4/10
WGJ80006	BA.2.75.6	222	membrane glycoprotein	2023/4/10
WGJ80007	BA.2.75.6	61	ORF6 protein	2023/4/10
WGJ80008	BA.2.75.6	121	ORF7a protein	2023/4/10
WGJ80009	BA.2.75.6	43	ORF7b protein	2023/4/10
WGJ80010	BA.2.75.6	121	ORF8 protein	2023/4/10
WGJ80011	BA.2.75.6	416	nucleocapsid phosphoprotein	2023/4/10
WGJ80012	BA.2.75.6	38	ORF10 protein	2023/4/10
WGH48577	BA.2.75.7	7093	ORF1ab polyprotein	2022/9/23
WGH48578	BA.2.75.7	4402	ORF1a polyprotein	2022/9/23
WGH48579	BA.2.75.7	1270	surface glycoprotein	2022/9/23
WGH48580	BA.2.75.7	275	ORF3a protein	2022/9/23
WGH48581	BA.2.75.7	75	envelope protein	2022/9/23
WGH48582	BA.2.75.7	222	membrane glycoprotein	2022/9/23
WGH48583	BA.2.75.7	61	ORF6 protein	2022/9/23
WGH48584	BA.2.75.7	121	ORF7a protein	2022/9/23
WGH48585	BA.2.75.7	43	ORF7b protein	2022/9/23
WGH48586	BA.2.75.7	121	ORF8 protein	2022/9/23
WGH48587	BA.2.75.7	416	nucleocapsid phosphoprotein	2022/9/23
WGH48588	BA.2.75.7	38	ORF10 protein	2022/9/23
WGG91140	CH.1.1.19	7093	ORF1ab polyprotein	2023/2/8
WGG91141	CH.1.1.19	4402	ORF1a polyprotein	2023/2/8
WGG91142	CH.1.1.19	1270	surface glycoprotein	2023/2/8
WGG91143	CH.1.1.19	275	ORF3a protein	2023/2/8
WGG91144	CH.1.1.19	75	envelope protein	2023/2/8
WGG91145	CH.1.1.19	222	membrane glycoprotein	2023/2/8
WGG91146	CH.1.1.19	61	ORF6 protein	2023/2/8
WGG91147	CH.1.1.19	121	ORF7a protein	2023/2/8
WGG91148	CH.1.1.19	43	ORF7b protein	2023/2/8
WGG91149	CH.1.1.19	121	ORF8 protein	2023/2/8
WGG91150	CH.1.1.19	416	nucleocapsid phosphoprotein	2023/2/8
WGG91151	CH.1.1.19	38	ORF10 protein	2023/2/8
WED18026	CH.1.1.6	7093	ORF1ab polyprotein	2023/2/21
WED18027	CH.1.1.6	4402	ORF1a polyprotein	2023/2/21
WED18028	CH.1.1.6	1270	surface glycoprotein	2023/2/21
WED18029	CH.1.1.6	275	ORF3a protein	2023/2/21
WED18030	CH.1.1.6	75	envelope protein	2023/2/21
WED18031	CH.1.1.6	222	membrane glycoprotein	2023/2/21
WED18032	CH.1.1.6	61	ORF6 protein	2023/2/21

WED18033	CH.1.1.6	121	ORF7a protein	2023/2/21
WED18034	CH.1.1.6	43	ORF7b protein	2023/2/21
WED18035	CH.1.1.6	121	ORF8 protein	2023/2/21
WED18036	CH.1.1.6	416	nucleocapsid phosphoprotein	2023/2/21
WED18037	CH.1.1.6	38	ORF10 protein	2023/2/21
WDT96447	P.1.4	7093	ORF1ab polyprotein	2021/7/5
WDT96448	P.1.4	4402	ORF1a polyprotein	2021/7/5
WDT96449	P.1.4	1273	surface glycoprotein	2021/7/5
WDT96450	P.1.4	275	ORF3a protein	2021/7/5
WDT96451	P.1.4	75	envelope protein	2021/7/5
WDT96452	P.1.4	222	membrane glycoprotein	2021/7/5
WDT96453	P.1.4	61	ORF6 protein	2021/7/5
WDT96454	P.1.4	121	ORF7a protein	2021/7/5
WDT96455	P.1.4	43	ORF7b protein	2021/7/5
WDT96456	P.1.4	121	ORF8 protein	2021/7/5
WDT96457	P.1.4	419	nucleocapsid phosphoprotein	2021/7/5
WDT96458	P.1.4	38	ORF10 protein	2021/7/5
WBO26258	B.1.617.1	7096	ORF1ab polyprotein	2021/4/9
WBO26259	B.1.617.1	4405	ORF1a polyprotein	2021/4/9
WBO26260	B.1.617.1	1273	surface glycoprotein	2021/4/9
WBO26261	B.1.617.1	275	ORF3a protein	2021/4/9
WBO26262	B.1.617.1	75	envelope protein	2021/4/9
WBO26263	B.1.617.1	222	membrane glycoprotein	2021/4/9
WBO26264	B.1.617.1	61	ORF6 protein	2021/4/9
WBO26265	B.1.617.1	121	ORF7a protein	2021/4/9
WBO26266	B.1.617.1	43	ORF7b protein	2021/4/9
WBO26267	B.1.617.1	121	ORF8 protein	2021/4/9
WBO26268	B.1.617.1	419	nucleocapsid phosphoprotein	2021/4/9
WBO26269	B.1.617.1	38	ORF10 protein	2021/4/9
WBK95201	P.1.13	7093	ORF1ab polyprotein	2021/6/21
WBK95202	P.1.13	4402	ORF1a polyprotein	2021/6/21
WBK95203	P.1.13	1273	surface glycoprotein	2021/6/21
WBK95204	P.1.13	275	ORF3a protein	2021/6/21
WBK95205	P.1.13	75	envelope protein	2021/6/21
WBK95206	P.1.13	222	membrane glycoprotein	2021/6/21
WBK95207	P.1.13	61	ORF6 protein	2021/6/21
WBK95208	P.1.13	121	ORF7a protein	2021/6/21
WBK95209	P.1.13	23	ORF7b protein	2021/6/21
WBK95210	P.1.13	50	ORF8 protein	2021/6/21
WBK95211	P.1.13	419	nucleocapsid phosphoprotein	2021/6/21
WBK95212	P.1.13	38	ORF10 protein	2021/6/21
UTE95752	C.37.1	7093	ORF1ab polyprotein	2021/5/5

UTE95753	C.37.1	4402	ORF1a polyprotein	2021/5/5
UTE95754	C.37.1	1266	surface glycoprotein	2021/5/5
UTE95755	C.37.1	275	ORF3a protein	2021/5/5
UTE95756	C.37.1	75	envelope protein	2021/5/5
UTE95757	C.37.1	222	membrane glycoprotein	2021/5/5
UTE95758	C.37.1	61	ORF6 protein	2021/5/5
UTE95759	C.37.1	121	ORF7a protein	2021/5/5
UTE95760	C.37.1	43	ORF7b protein	2021/5/5
UTE95761	C.37.1	121	ORF8 protein	2021/5/5
UTE95762	C.37.1	419	nucleocapsid phosphoprotein	2021/5/5
UTE95763	C.37.1	38	ORF10 protein	2021/5/5
UOM13148	B.1.351.3	7093	ORF1ab polyprotein	2021/5/31
UOM13149	B.1.351.3	4402	ORF1a polyprotein	2021/5/31
UOM13150	B.1.351.3	1270	surface glycoprotein	2021/5/31
UOM13151	B.1.351.3	275	ORF3a protein	2021/5/31
UOM13152	B.1.351.3	75	envelope protein	2021/5/31
UOM13153	B.1.351.3	222	membrane glycoprotein	2021/5/31
UOM13154	B.1.351.3	61	ORF6 protein	2021/5/31
UOM13155	B.1.351.3	121	ORF7a protein	2021/5/31
UOM13156	B.1.351.3	43	ORF7b protein	2021/5/31
UOM13157	B.1.351.3	121	ORF8 protein	2021/5/31
UOM13158	B.1.351.3	419	nucleocapsid phosphoprotein	2021/5/31
UOM13159	B.1.351.3	38	ORF10 protein	2021/5/31
UZY79434	BA.2.75.9	7093	ORF1ab polyprotein	2022/9/29
UZY79435	BA.2.75.9	4402	ORF1a polyprotein	2022/9/29
UZY79436	BA.2.75.9	1270	surface glycoprotein	2022/9/29
UZY79437	BA.2.75.9	275	ORF3a protein	2022/9/29
UZY79438	BA.2.75.9	75	envelope protein	2022/9/29
UZY79439	BA.2.75.9	222	membrane glycoprotein	2022/9/29
UZY79440	BA.2.75.9	61	ORF6 protein	2022/9/29
UZY79441	BA.2.75.9	121	ORF7a protein	2022/9/29
UZY79442	BA.2.75.9	43	ORF7b protein	2022/9/29
UZY79443	BA.2.75.9	121	ORF8 protein	2022/9/29
UZY79444	BA.2.75.9	416	nucleocapsid phosphoprotein	2022/9/29
UZY79445	BA.2.75.9	38	ORF10 protein	2022/9/29
UYR40138	BA.2.75.8	7093	ORF1ab polyprotein	2022/10/16
UYR40139	BA.2.75.8	4402	ORF1a polyprotein	2022/10/16
UYR40140	BA.2.75.8	1270	surface glycoprotein	2022/10/16
UYR40141	BA.2.75.8	275	ORF3a protein	2022/10/16
UYR40142	BA.2.75.8	75	envelope protein	2022/10/16
UYR40143	BA.2.75.8	222	membrane glycoprotein	2022/10/16
UYR40144	BA.2.75.8	61	ORF6 protein	2022/10/16

UYR40145	BA.2.75.8	121	ORF7a protein	2022/10/16
UYR40146	BA.2.75.8	43	ORF7b protein	2022/10/16
UYR40147	BA.2.75.8	121	ORF8 protein	2022/10/16
UYR40148	BA.2.75.8	416	nucleocapsid phosphoprotein	2022/10/16
UYR40149	BA.2.75.8	38	ORF10 protein	2022/10/16
UUL68129	B.1.640	7075	ORF1ab polyprotein	2022/1/8
UUL68130	B.1.640	4384	ORF1a polyprotein	2022/1/8
UUL68131	B.1.640	1273	surface glycoprotein	2022/1/8
UUL68132	B.1.640	275	ORF3a protein	2022/1/8
UUL68133	B.1.640	75	envelope protein	2022/1/8
UUL68134	B.1.640	222	membrane glycoprotein	2022/1/8
UUL68135	B.1.640	61	ORF6 protein	2022/1/8
UUL68136	B.1.640	121	ORF7a protein	2022/1/8
UUL68137	B.1.640	29	ORF7b protein	2022/1/8
UUL68138	B.1.640	419	nucleocapsid phosphoprotein	2022/1/8
UUL68139	B.1.640	38	ORF10 protein	2022/1/8

Table S3. The immune response antigens of neutralizing antibodies.

SARS-CoV-2 antigen	Neutralizing assay for linear epitopes	Other antibody response assay for linear epitopes	Neutralizing assay for discontinuous epitopes	Other antibody response assay for discontinuous epitopes
Spike	117	2253	1028	218
ORF1ab	0	3751	0	1
ORF3a	0	3392	0	1
E	0	42	0	0
M	1	202	0	0
ORF6	0	33	0	0
ORF7a	0	60	0	0
ORF7b	0	2	0	0
ORF8	0	97	0	0
N	1	811	1	3
ORF10	0	25	0	0

Table S4. Linear B-cell candidate epitopes for the pre-emptive Pan-Coronavirus Vaccine as well as toxin prediction for each epitope.

Epitopes	IEDB ID	sequence	protein	position	RF scores (95% CI)	Assay Description	Toxin
B1	1336532	DISTEIYQAGSTPCNGVEGFNCYFPL QSYGFQPTNGVGYQPYRVVVL	Spike glycoprotein	467-513	1.00 (0.04:1.00)	biological activity/neutralization/Positive	Non-toxin
B2	1071808	PSKPSKRSFIEDLLFNKV	Spike glycoprotein	809-826	0.90 (0.60:0.99)	biological activity/neutralization/Positive-Low	Non-toxin
B3	2001237	PPLLTDEMIAQYTSA	Spike glycoprotein	862-876	0.51(0.37:0.65)	biological activity/neutralization/Positive	Non-toxin
B4	1386752	PLQPELDSFKEELDKYFKNHTSPDVDL GDISGIN	Spike glycoprotein	1140-1173	0.12(0.07:0.22)	biological activity/neutralization/Positive	Non-toxin
B5	1313131	MADSNGTITVEELKK	Membrane protein	1-15	0.28 (0.21:0.37)	ICS/IFNg release/Positive/biological activity/neutralization/Positive	Non-toxin
B6	1392375	QGTTLPKGFYAEGSRGGS	Nucleoprotein	163- 180	1.00 (0.04:1.00)	biological activity/neutralization/Positive	Non-toxin
B7	1391530	YLTPGDSSSGWTAGAAAYYV	surface glycoprotein	248-267	1.00（0.47：1.00）	ICS/IFNg/IL-17/IL-2 release/Positive/biological activity/neutralization/Positive	Non-toxin
B8	1073281	TESNKKFLPFQQFGRDIA	surface glycoprotein	556-570	0.78 (0.71:0.84)	ELISA/qualitative binding/Positive	Non-toxin
B9	1334467	KVGGNYNYLYRLFRKSNLKPFERDIS	surface glycoprotein	444-469	0.15 (0.10:0.24)	biological activity/neutralization/Positive-High	Non-toxin
B10	1334470	NNLDSKVGGNYNYLYR	surface glycoprotein	439-454	0.05 (0.02:0.14)	biological activity/neutralization/Positive-High	Non-toxin
B11	1309518	LNEVAKNLNESLIDLQELGK	surface glycoprotein	1186-1205	0.04 (0.02:0.09)	biological activity/neutralization/Positive	Non-toxin
B12	1575456	IRGDEVRQIAPGQTGKIADYNYK	surface glycoprotein	402-424	1.00 (0.04:1.00)	biological activity/neutralization/Positive	Non-toxin

Table S5. The candidate immunodominant B-cell epitopes exhibited a high degree of conservation among SARS-CoV-2 VOCs and VOIs.

Epitopes	Sequence	conservancy
B1(WT)	DISTEIYQAGSTPCNGVEGFNCYFPLQSYGFQPTNGVG YQPYRVVVL	100%
B1（variant1）	DISTEIYQAGSTPCNGVKGFNCYFPLQSYGFQPTYGVG YQPYRVVVL	95.74%
B1（variant2）	DISTEIYQAGSTPCNGVEGFNCYSPLQSYGFQPTNGVG YQPYRVVVL	97.87%
B1（variant3）	DISTEIYQAGNKPCNGVAGFNCYFPLQSYGFRPTYGV GHQPYRVVVL	87.23%
B1（variant4）	DISTEIYQAGNKPCNGVAGSNCYFPLQSYGFRPTYGV GHQPYRVVVL	85.11%
B1（variant5）	DISTEIYQAGSTPCNGVQGFNCYFPLQSYGFQPTNGVG YQPYRVVVL	97.87%
B1（variant6）	DISTEIYQAGNKPCNGVAGSNCYFPLQSYGFRPTYGV GYQPYRVVVL	87.23%
B1（variant7）	DISTEIYQAGNRPCNGVAGPNCYSPLQSYGFRPTYGVG HQPYRVVVL	82.98%
B1（variant8）	DISTEIYQAGSTPCNGVEGFNCYFPLQSYGFQPTYGVG YQPYRVVVL	97.87%
B1（variant9）	DISTEIYQAGNRPCNGVAGSNCYFPLQSYGFRPTYGVG HQPYRVVVL	85.11%
B1（variant10）	DISTEIYQAGNKPCNGVAGVNCYFPLQSYGFRPTYGV GHQPYRVVVL	85.11%
B1（variant11）	DISTEIYQAGSKPCNGVEGFNCYFPLQSYGFQPTNGVG YQPYRVVVL	97.87%
B1（variant12）	DISTEIYQAGNKPCNGVAGPNCYSPLQSYGFRPTYGV GHQPYRVVVL	82.98%
B1（variant13）	DISTEIYQAGSKPCNGVEGFNCYFPLQSYGFQPTYGVG YQPYRVVVL	95.74%
B1（variant14）	DISTEIYQAGNKPCNGVAGFNCYFPLRSYSFRPTYGVG HQPYRVVVL	82.98%
B1（variant15）	DISTEIYQAGSTPCNGVKGFNCYFPLQSYGFQPTNGVG YQPYRVVVL	97.87%
B1（variant16）	DISTEIYQAGNNPCNGVAGPNCYSPLQSYGFRPTYGV GHQPYRVVVL	82.98%
B1（variant17）	DISTEIYQAGNKPCNGVAGPNCYSPLQPYGFRPTYGV GHQPYRVVVL	80.85%
B1（variant18）	DISTEIYQAGNQPCNGVAGPNCYSPLQSYGFRPTYGV GHQPYRVVVL	82.98%
B1（variant19）	DISTEIYQAGNKPCNGVAGFNCYFPLRSYGFRPTYGVG HQPYRVVVL	85.11%

B2(WT)	PSKPSKRSFIEDLLFNKV	100%
B2(variant1)	PSKPSKRSFIEDLLFNKL	94.44%
B2(variant2)	PSKSSKRSFIEDLLFNKV	94.44%
B4(WT)	PLQPELDSFKEELDKYFKNHTSPDVDLGDISGIN	100%
B4(variant1)	PLQLELDSFKEELDKYFKNHTSPDVDLGDISGIN	97.06%
B5(WT)	MADSNGTITVEELKK	100%
B5(variant1)	MTDSNGTITVEELKK	93.33%
B5(variant2)	MANSNGTITVEELKK	93.33%
B5(variant3)	MAGSNGTITVEELKK	93.33%
B6(WT)	QGTTLPKGFYAEGSRGGS	100%
B6(variant1)	QGTTLPKGFYVEGSRGGS	94.44%
B7(WT)	YLTPGDSSSGWTAGAAAYYV	100%
B7(variant1)	YLTPGDSSSSWTAGAAAYYV	95.00%
B7(variant2)	YLTPVDSSSGWTAGAAAYYV	95.00%
B7(variant3)	YLTPGGSSSGWTAGAAAYYV	95.00%
B7(variant4)	NSTPGDSSSGWTAGAAAYYV	90.00%
B7(variant5)	YLTPGGSSLGWTAGAAAYYV	90.00%
B8(WT)	TESNKKFLPFQQFGRDIA	100%
B8(variant1)	TESNKKFLPFQQFGRDID	94.44%
B8(variant2)	TASNKKFLPFQQFGRDIA	94.44%
B8(variant3)	TKSNKKFLPFQQFGRDIA	94.44%
B9(WT)	KVGGNYNYLYRLFRKSNLKPFERDIS	100%
B9(variant1)	KVGGNYNYQYRLFRKSNLKPFERDIS	96.15%
B9(variant2)	KVSGNYNYQYRLFRKSKLKPFERDIS	88.46%
B9(variant3)	KVSGNYNYLYRLFRKSKLKPFERDIS	92.31%
B9(variant4)	KVGGNYNYRYRLFRKSNLKPFERDIS	96.15%
B9(variant5)	TVSGNYNYRYRLFRKSKLKPFERDIS	84.62%
B9(variant6)	KPSGNYNYLYRLFRKSKLKPFERDIS	88.46%
B9(variant7)	KVSGNYNYRYRLFRKSKLKPFERDIS	88.46%
B9(variant8)	TVGGNYNYRYRLFRKSKLKPFERDIS	88.46%
B9(variant9)	KVGGNYNYRYRLFRKSNLKSFERDIS	92.31%
B9(variant10)	KVSGNYNYLYRLFRKSNLKPFERDIS	96.15%
B9(variant11)	KPSGNYNYLYRLLRKSKLKPFERDIS	84.62%
B10(WT)	NNLDSKVGGNYNYLYR	100%
B10(variant1)	NNLDSKVGGNYNYQYR	93.75%
B10(variant2)	NNLDSKVGGNNNYLYR	93.75%
B10(variant3)	NKLDSKVSGNYNYQYR	81.25%
B10(variant4)	NKLDSKVSGNYNYLYR	87.50%
B10(variant5)	NNLDSKVGGNYNYRYR	93.75%
B10(variant6)	NKLDSTVSGNYNYRYR	75.00%
B10(variant7)	NKLDSKPSGNYNYLYR	81.25%
B10(variant8)	NKLDSKVSGNYNYRYR	81.25%

B10(variant9)	NKLDSTVGGNYNYRYR	81.25%
B10(variant10)	NKLDSKVGGNYNYRYR	87.50%
B10(variant11)	NKLDSKVGGNYNYQYR	87.50%
B10(variant12)	NKLDSKVGGNYNYLYR	93.75%
B11(WT)	LNEVAKNLNESLIDLQELGK	100%
B11(variant1)	LNEVAKNLNESLINLQELGK	95.00%
B12(WT)	IRGDEVRQIAPGQTGKIADYNYK	100%
B12(variant1)	IRGDEVRQIAPGQTGNIADYNYK	95.65%
B12(variant2)	IRGNEVSQIAPGQTGNIADYNYK	86.96%
B12(variant3)	IRGDEVRQIAPGQTGTIADYNYK	95.65%
B12(variant4)	IRGNEVSQIAPGQTGNIADFNYK	82.61%

Table S6. The candidate immunodominant B-cell epitopes exhibited a high degree of conservation among zoonotic coronaviruses.

Epitopes	Sequence	conservancy	virus ID
B1(WT)	DISTEIYQAGSTPCNGVEGFNCYFPLQSYGFQPTNGVGYQPYRVVVL	100%	QHD43416
B1(variant1)	DISTEIYQAGSKPCNGQTGLNCYYPLYRYGFYPTDGVGHQPYRVVVL	78.72%	MN996532
B1(variant2)	DISTEIYQAGSTPCNGQVGLNCYYPLERYGFHPTTGVNYQPFRVVVL	78.72%	MT040333
B1(variant3)	DISTEIYQAGSTPCNGVEGFNCYFPLQSYGFHPTNGVGYQPYRVVVL	97.87%	MT121216
B2(WT)	PSKPSKRSFIEDLLFNKV	100%	QHD43416, MN996532, MT040333, MT040334, MT040335, MT040336, MT072864, MT121216
B2(variant1)	PLKPTKRSFIEDLLFNKV	88.89%	AAP13441, AY572034, KF367457, KT444582
B2(variant2)	CSKASSRSAIEDLLFDKV	72.22%	AIV41879
B2(variant3)	PSKPTKRSFIEDLLFNKV	94.44%	FJ211859, FJ588686
B2(variant4)	PLKPTKRSFIEDLLYNKV	83.33%	KP886808
B3(WT)	PPLLTDEMIAQYTSA	100%	QHD43416, MN996532, MT040333, MT040334, MT040335, MT040336, MT072864, MT121216,
B3(variant1)	PPLLTDDMIAAYTAA	80.00%	AAP13441, AY572034, KF367457, KP886808, KT444582
B3(variant2)	PPLLTDEMVAAYTAA	80.00%	FJ211859
B3(variant3)	PPLLTDEMIAAYTAA	86.67%	FJ588686
B4(WT)	PLQPELDSFKEELDKYFKNHTSPDVDLGDISGIN	100%	AAP13441, QHD43416, AY572034, FJ211859, FJ588686, KF367457, KP886808, KT444582,

			MN996532, MT040333, MT040334, MT040335, MT040336, MT072864, MT121216,
B5(WT)	MADSNGTITVEELKK	100%	QHD43419
B5(variant1)	MADNSTITVEELKQ	64.29%	AY572034
B5(variant2)	MAENGTISVEELKR	64.29%	lFJ588686, KF367457, KP886808, KT444582
B5(variant3)	MADNGTITVEELKK	78.57%	MN996532
B5(variant4)	SADNGTITVEELKK	78.57%	MT040333, MT040334, MT040335, lMT040336
B5(variant5)	SANNGTITVEELKK	78.57%	MT072864
B5(variant6)	SGDNGTITVEELKK	78.57%	MT121216
B6(WT)	QGTTLPKGFYAEGSRGGS	100%	AAP13445, AY572034, FJ211859, FJ588686, KF367457, KP886808,KT444582, MN996532, MT121216,
B6(variant1)	PGTELPKGFYVEGSRSTS	72.22%	KF636752
B6(variant2)	QGTALPKGFYAEGSRGGS	94.44%	MT040333, MT040334, MT040335, MT040336, MT072864
B7(WT)	YLTPGDSSSGWTAGAAAYYV	100%	QHD43416, MN996532
B7(variant1)	YLTPGNLESGWTTGAAAYYV	80.00%	MT040333
B7(variant2)	YLTPGKLESGWTTGAAAYYV	80.00%	lMT040334,MT040335, MT040336, MT072864
B8(WT)	TESNKKFLPFQQFGRDIA	100%	QHD43416
B9(WT)	KVGGNYNYLYRLFRKSNLKPFERDIS	100%	QHD43416, MT121216,
B9(variant1)	KEGGNFNYLYRLFRKANLKPFERDI	88.00%	MN996532
B9(variant2)	LTGGNYGYLYRLFRKSKLKPFERDI	84.00%	MT040333, MT040335, MT072864
B9(variant3)	LTGDNYGYLYRLFRKSKLKPFERDI	80.00%	MT040336
B10(WT)	NNLDSKVGGNYNYLYR	100%	QHD43416, MT121216,
B11(WT)	LNEVAKNLNESLIDLQELGK	100%	AAP13441, QHD43416, AY572034, FJ211859, FJ588686, KF367457, KT444582, MN996532,

			MT040334, MT040335, MT040336, MT072864, MT121216,
B11(variant1)	LQEVVKQLNDSYIDLKELGN	65.00%	EF065505
B11(variant2)	LQQVVKALNESYIDLKELGN	65.00%	KF917527, NC_019843
B11(variant3)	LSEVVKQLNESYIDLKELGN	70.00%	MH002342
B11(variant4)	LNEVAKNLNESPIDLQELGK	95.00%	MT040333
B11(variant5)	LTDVVNQLNQSYIDLKQLGT	50.00%	MT350598
B12(WT)	IRGDEVRQIAPGQTGKIADYNYK	100%	QHD43416
B12(variant1)	IRSSEVRQVAPGETGVIADYNYK	78.26%	FJ211859, FJ588686
B12(variant2)	VKGDDVRQIAPGQTGVIADYNYK	82.61%	AAP13441, KF367457, lKT444582
B12(variant3)	IRFSEVRQVAPGQTGVIADYNYK	82.61%	KP886808
B12(variant4)	ITGDEVRQIAPGQTGKIADYNYK	95.65%	MN996532
B12(variant5)	VKGDEVRQIAPGQTGVIADYNYK	86.96%	MT040333, MT040334, MT040335, MT040336, MT072864
B12(variant6)	VRGDEVRQIAPGQTGRIADYNYK	91.30%	MT121216

Table S7. Helper T-lymphocyte specific epitope for the candidate pre-emptive Pan-Coronavirus Vaccine as well as toxin prediction for each epitope.

Epitope	IEDB ID	Sequence	Protein	RF Score (95% CI)	MHC restriction	Assay description	Toxin
**Th1**	**1310622**	**LSYYKLGASQRVAGD**	**Membrane**	0.76 (0.71:0.80)	**HLA class I;HLA class II;HLA-DPB1*02:01;HLA-DQA1*01:01/DQB1*05:01;HLA-DQA1*01:02/DQB1*06:02;HLA-DQA1*05:01/DQB1*02:01;HLA-DQA1*05:01/DQB1*03:01;HLA-DQB1*02:01;HLA-DQB1*03:01;HLA-DQB1*06:02;HLA-DQB1*06:03;HLA-DRB1*01:01;HLA-DRB1*03:01;HLA-DRB1*04:01;HLA-DRB1*04:05;HLA-DRB1*07:01;HLA-DRB1*08:02;HLA-DRB1*09:01;HLA-DRB1*11:01;HLA-DRB1*12:01;HLA-DRB1*13:02;HLA-DRB1*14:06;HLA-DRB1*14:06;HLA-DRB1*15:01;HLA-DRB1*16:01;HLA-DRB1*16:02;HLA-DRB3*01:01;HLA-DRB3*02:02;HLA-DRB4*01:01;HLA-DRB5*01:01**	**IFNg release/TNF release/activation/ qualitative binding/degranulationTNFa release**	**Non-toxin**
**Th2**	**1310598**	**LLLLDRLNQLESKMS**	**Nucleoprotein**	0.38 (0.33:0.43)	**HLA class II;HLA-DPA1*01:03/DPB1*04:01;HLA-DPB1*02:01;HLA-DQA1*01:01/DQB1*05:01;HLA-DQA1*01:02/DQB1*06:02;HLA-DQA1*03:01/DQB1*03:02;HLA-DQA1*05:01/DQB1*02:01;HLA-DR;HLA-DRB1*01:01;HLA-DRB1*03:01;HLA-DRB1*04:01;HLA-DRB1*04:05;HLA-DRB1*07:01;HLA-DRB1*08:02;HLA-DRB1*09:01;HLA-DRB1*11:01;HLA-DRB1*12:01;HLA-DRB1*13:02;HLA-DRB1*14:01;HLA-DRB1*15:01;HLA-DRB3*01:01;HLA-DRB4*01:01;HLA-DRB5*01:01**	**IFNg release/TNF release/activation/IL-2 release**	**Non-toxin**
**Th3**	**1309110**	**CTFEYVSQPFLMDLE**	**Spike**	-	**HLA class I;HLA class II;HLA-C*07:02;HLA-DQB1*02:01;HLA-DQB1*02:02;HLA-DQB1*05:02;HLA-DQB1*05:03;HLA-DR4;HLA-DRB1;HLA-DRB1*01:01;HLA-DRB1*03:01;HLA-DRB1*04:05;HLA-DRB1*07:01;HLA-DRB1*09:01;HLA-DRB1*12:01;HLA-DRB1*16:01**	**IFNg release/IL-5 release/activation/qualitative binding/activation**	**Non-toxin**
**Th4**	**1309132**	**NFSQILPDPSKPSKR**	**Spike**	-	**HLA-DQA1*03:02; HLA-DQA1*05:01; HLA-DQA1*01:01;HLA-DQA1*05:05; HLA-DQA1*02:01;HLA-DQA1*01:02; HLA-DQB1*02:01;HLA-DQB1*02:02; HLA-DQB1*03:01; HLA-DQB1*05:01; HLA-DQB1*06:02; HLA-DRB1*03:01; HLA-DRB1*04:05; HLA-DRB1*01:01; HLA-DRB1*11:01; HLA-DRB1*07:01;HLA-DRB1*15:01**	**IFNg release/activation/IL-5 release/IL-13 release/IL-10 release/TNFa release/IL-4 release**	**Non-toxin**
**Th5**	**1310286**	**ASAFFGMSRIGMEV**	**Nucleo**	0.31	**HLA class II;HLA-DQB1*05:03;HLA-DR;HLA-DR11;HLA-DRB1*01:02;HLA-**	**IFNg release/TNF**	**Non-toxin**

		T		(0.23:0.41)	DRB1*11:01;HLA-DRB1*11:04;HLA-DRB1*14:01	release/activation/IL-2 release	
**Th6**	**1310430**	**FYVYSRVKNLNSSRV**	**Envelope**	0.55 (0.48:0.62)	**HLA class II;HLA-DR;HLA-DR11; HLA-DRB1*01:01;HLA-DRB1*04:01;HLA-DRB1*15:01**	**IFNg release/TNF release/qualitative binding/IFNg release**	**Non-toxin**
**Th7**	**1071580**	**NLLLQYGSFCTQLNR**	**Spike**	0.19 (0.14:0.25)	**HLA class I;HLA class II;HLA-DQB1*05:03;HLA-DRB1*04:04;HLA-DRB1*15:01**	**IFNg release/IL-5 release/activation/IIFNg release**	**Non-toxin**
**Th8**	**1131160**	**DDQIGYYRRATRRIR**	**Nucleoprotein**	0.22 (0.16:0.29)	**HLA class II;HLA-DPB1*02:01;HLA-DQA1*05:01/DQB1*03:01;HLA-DRB1*01:01;HLA-DRB1*04:01;HLA-DRB1*04:05;HLA-DRB1*07:01;HLA-DRB1*08:02;HLA-DRB1*09:01;HLA-DRB1*11:01;HLA-DRB1*13:01;HLA-DRB1*14:01;HLA-DRB1*14:06;HLA-DRB1*15:01;HLA-DRB1*16:02;HLA-DRB3*01:01;HLA-DRB3*02:02;HLA-DRB5*01:01**	**IL-5 release/IFNg release/TNFa release/activation**	**Non-toxin**
**Th9**	**1309140**	**TDEMIAQYTSALLAG**	**Spike**	0.45 (0.36:0.55)	**HLA class II;HLA-DRB1*04:04;HLA-DRB1*15:01;HLA-DQB1*06:02**	**IFNg release/activation**	**Non-toxin**
**Th10**	**1310796**	**SFIEDLLFNKVTLAD**	**Spike**	0.45 (0.36:0.55)	**HLA class I;HLA class II;HLA-DQB1*05:03;HLA-DRB1;HLA-DRB1*03:01;HLA-DRB1*12:01;HLA-DRB1*13:02;HLA-DRB1*14:01**	**IFNg release/IL-5 release/activation、proliferation**	**Non-toxin**
**Th11**	**1310806**	**SKWYIRVGARKSAPL**	**ORF8 protein**	0.78 (0.70:0.83)	**H2-d class I;HLA class II;HLA-DR;HLA-DR4**	**IFNg release、TNF release、qualitative binding**	**Non-toxin**
**Th12**	**1310865**	**TRFQTLLALHRSYLT**	**Spike**	0.34 (0.26:0.43)	**HLA class I;HLA class II;HLA-DQB1*05:03;HLA-DRB1;HLA-DRB1*01:01;HLA-DRB1*04:01;HLA-DRB1*04:05;HLA-DRB1*07:01;HLA-DRB1*08:02;HLA-DRB1*09:01;HLA-DRB1*11:01;HLA-DRB1*12:01;HLA-DRB1*14:01;HLA-DRB1*15:01;HLA-DRB1*15:02**	**IFNg release/IL-5 release/activation/qualitative binding**	**Non-toxin**
**Th13**	**1310292**	**ASWFTALTQHGKEDL**	**Nucleoprotein**	0.46 (0.39:0.53)	**H2 class I;HLA class II;HLA-DR;HLA-DRB4*01:03**	**IFNg release**	**Non-toxin**
**Th14**	**1310488**	**IGYYRRATRRIRGGD**	**Nucleoprotein**	0.60 (0.49:0.70)	**HLA class II;HLA-DR;HLA-DR11;HLA-DRB1*11:01;HLA-DRB1*11:04;HLA-DRB1*13:01**	**IFNg release/TNF release/activation/IL-2 release**	**Non-toxin**
**Th15**	**1310503**	**IPFAMQMAYRFNGIG**	**Spike**	-	**HLA class I;HLA class II;HLA-DQB1*04:02;HLA-DQB1*05:03;HLA-DRB1;HLA-DRB1*01:01;HLA-DRB1*04:01;HLA-DRB1*07:01;HLA-DRB1*08:02;HLA-DRB1*09:01;HLA-DRB1*11:01;HLA-DRB1*12:01;HLA-DRB1*14:01;HLA-DRB1*15:01**	**IFNg release/activation/qualitative binding**	**Non-toxin**

**Th16**	**1310780**	**RWYFYYLGTGPEAGL**	**Nucleoprotein**	0.41 (0.32:0.51)	**HLA-DR;HLA class II;HLA-DRB1*11:01;HLA-DRB3*02:02**	**IFNg release/TNF release/activation/IL-2 release**	**Non-toxin**

Table S8. The candidate immunodominant Th-cell epitopes exhibited a high degree of conservation among SARS-CoV-2 VOCs and VOIs.

Epitopes	Sequence	conservancy
Th2(WT)	LLLLDRLNQLESKMS	100%
Th2(variant1)	LLLLDRLNQLESKMF	93.33%
Th2(variant2)	LLLLDRLNQLENKMS	93.33%
Th3(WT)	CTFEYVSQPFLMDLE	100%
Th3(variant1)	CTFEYVSQPFLMDLV	93.33%
Th4(WT)	NFSQILPDPSKPSKR	100%
Th4(variant1)	NFSQILPDPSKSSKR	93.33%
Th6(WT)	FYVYSRVKNLNSSRV	100%
Th6(variant1)	IYVYSRVKNLNSSRV	93.33%
Th6(variant2)	FYVYSRVKNLNSFRV	93.33%
Th7(WT)	NLLLQYGSFCTQLNR	100%
Th7(variant1)	NLLLQYGSFCTQLKR	93.33%
Th8(WT)	DDQIGYYRRATRRIR	100%
Th8(variant1)	DDQIGYYRRSTRRIR	93.33%
Th10(WT)	SFIEDLLFNKVTLAD	100%
Th10(variant1)	SFIEDLLFNKLTLAD	93.33%
Th12(WT)	TRFQTLLALHRSYLT	100%
Th12(variant1)	TRFQTLLALHRRNST	80.00%
Th12(variant2)	TRFQTLLALYRSYLT	93.33%
Th13(WT)	ASWFTALTQHGKEDL	100%
Th13(variant1)	ASWFTALTQHGKEGL	93.33%
Th14(WT)	IGYYRRATRRIRGGD	100%
Th14(variant1)	IGYYRRSTRRIRGGD	93.33%

Table S9. The candidate immunodominant Th-cell epitopes exhibited a high degree of conservation among zoonotic coronaviruses.

Epitopes	Sequence	conservancy	ID
Th1(WT)	LSYYKLGASQRVAGD	100%	QHD43419,MN996532, MT040333, MT040334, MT040335, MT040336, MT072864, MT121216
Th1(variant1)	LSYYKLGASQRVGTD	86.67%	AAP13444, AY572034,FJ211859, FJ588686, KF367457, KP886808, KT444582
Th2(WT)	LLLLDRLNQLESKMS	100%	QHD43423
TH2(variant1)	LLLLDRLNQLESKVS	93.33%	AAP13445,AY572034, FJ211859, FJ588686, KF367457, KP886808, KT444582
TH2(variant2)	LLLLDRLNALESKMS	93.33%	MT040333, MT040334, MT040335, MT040336, MT072864
Th3(WT)	CTFEYVSQPFLMDLE	100%	QHD43416, MN996532
Th3(variant1)	CTFEYISQPFLMDLE	93.33%	MT040333, MT040334, MT040335, MT040336, MT072864
Th4(WT)	NFSQILPDPSKPSKR	100%	QHD43416, MN996532, MT040333, MT040334, MT040335, MT040336, MT121216,
Th4(variant1)	NFSQILPDPLKPTKR	86.67%	AAP13441, AY572034, KF367457, KP886808, KT444582
Th4(variant2)	NFSQILPDPSKPTKR	93.33%	FJ211859, FJ588686
Th4(variant3)	NFLQILPDPSKPSKR	93.33%	MT072864
Th5(WT)	ASAFFGMSRIGMEVT	100%	AAP13445, AY572034, FJ211859, FJ588686, KF367457, KP886808,KT444582, MN996532, MT040333, MT040334, MT040335, MT040336, MT072864, MT121216,
Th6(WT)	FYVYSRVKNLNSSRV	100%	QHD43416
Th7(WT)	NLLLQYGSFCTQLNR	100%	AAP13441, QHD43416, FJ211859, FJ588686, KF367457, KP886808, KT444582, MN996532, MT040333, MT040334, MT040335, MT040336, MT072864, MT121216,
Th7(variant1)	NLLLQYGSFCRQLNR	93.33%	AY572034
Th8(WT)	DDQIGYYRRATRRIR	100%	QHD43423, MN996532, MT121216,
Th8(variant1)	DDQIGYYRRATRRVR	93.33%	AAP13445, AY572034, FJ211859, FJ588686, KF367457, KP886808, KT444582, MT040333, MT040334, MT040335, MT040336, MT072864
Th9(WT)	TDEMIAQYTSALLAG	100%	QHD43416, MN996532, MT040333, MT040334, MT040335, MT040336, MT072864, MT121216,
Th9(variant1)	TDEMVAAYTAALVSG	66.67%	FJ211859
Th9(variant2)	TDDMIAAYTAALVSG	66.67%	AAP13441, AY572034, KF367457, KT444582
Th9(variant3)	TDEMIAAYTAALVSG	73.33%	FJ588686

Th9(variant4)	TDDMIAAYTAALISG	66.67%	KP886808
Th10(WT)	SFIEDLLFNKVTLAD	100%	AAP13441, QHD43416, AY572034, FJ211859, FJ588686, KF367457, KT444582, MN996532, MT040333, MT040334, MT040335, MT040336, MT072864, MT121216,
Th10(variant1)	SLLEDLLFNKVKLSD	73.33%	AAX76521
Th10(variant2)	SAIEDLLFDKVKLSD	73.33%	AIV41879, FJ647226, KT368891, MN514967
Th10(variant3)	SAIEDLLFDKVTIAD	80.00%	EF065505, KF917527,NC_019843
Th10(variant4)	SAIEDILFNKVKLSD	73.33%	KM349742
Th10(variant5)	SFIEDLLYNKVTLAD	93.33%	KP886808
Th10(variant6)	SAIEDLLFNKVTIAD	86.67%	MH002342
Th10(variant7)	SAISDLLYNKVKVAD	66.67%	MT350598
Th11(WT)	SKWYIRVGARKSAPL	100%	QHD43422, MT040333, MT040335, MT040336, MT072864
Th11(variant1)	SRWFIRVGARKSAPL	86.67%	MT121216
Th12(WT)	KYYTVIPRSFRSKAN	100%	EF065505
Th12(variant1)	LYYTVIPRSIRSPFN	73.33%	MH002342
Th13(WT)	ASWFTALTQHGKEDL	100%	QHD43423, MN996532, MT040333, MT040334, MT040335, MT040336, MT072864, MT121216,
Th13(variant1)	ASWFTALTQHGKEEL	93.33%	AAP13445, AY572034, FJ211859, FJ588686, KF367457, KP886808, KT444582
Th13(variant2)	VSWYTGLTQHGKNPL	66.67%	EF065505
Th13(variant3)	VSWFTPLTQHGKQAL	73.33%	KF636752
Th13(variant4)	VSWYTGLTQHGKVPL	66.67%	KF917527, NC_019843
Th13(variant5)	VSWFAPLVQTGKQDL	60.00%	MG762674
Th13(variant6)	VSWFTGLTQHGKQPL	73.33%	MH002342
Th14(WT)	IGYYRRATRRIRGGD	100%	QHD43423, MN996532, MT121216,
Th14(variant1)	IGYYRRATRRVRGGD	93.33%	AAP13445, AY572034, FJ211859, FJ588686, KF367457, KP886808, KT444582, MT040333, MT040334, MT040335, MT040336, MT072864
Th15(WT)	IPFAMQMAYRFNGIG	100%	AAP13441, QHD43416, AY572034, FJ211859, FJ588686, KF367457, KP886808, KT444582, MN996532, MT040333, MT040334, MT040335, MT040336, MT072864, MT121216,
Th15(variant1)	IPNTMQMAYRFNGIA	80.00%	KF636752
Th16(WT)	RWYFYYLGTGPEAGL	100%	QHD43423, MN996532, MT040333, MT040334, MT040335, MT040336, MT072864, MT121216,
Th16(variant1)	RWYFYYLGTGPEASL	93.33%	AAP13445, AY572034, FJ211859, FJ588686, KF367457, KP886808, KT444582
Th16(variant2)	RWYFYYLGTGPYASS	80.00%	AAX76525

**Th16(variant3)**	**RWYFYYLGTGPHAKD**	**80.00%**	**AIV41880, MN514967**
**Th16(variant4)**	**RWFFYYTGTGPEANL**	**80.00%**	**EF065505**
**Th16(variant5)**	**RWYFYYLGTGPYAGA**	**86.67%**	**FJ647226**
**Th16(variant6)**	**RWYFYYLGTGPEQNL**	**86.67%**	**KF636752**
**Th16(variant7)**	**RWYFYYTGTGPEAAL**	**86.67%**	**KF917527, NC_019843**
**Th16(variant8)**	**RWYFYYLGTGPYANK**	**80.00%**	**KM349742**
**Th16(variant9)**	**RWYFYYTGTGRFGDL**	**66.67%**	**MG762674**
**Th16(variant10)**	**RWYFYYTGTGPEANL**	**86.67%**	**MH002342**
**Th16(variant11)**	**RWYFYYTGTGRYADL**	**73.33%**	**MT350598**

Table S10. Cytotoxic T-lymphocyte specific epitope for the candidate pre-emptive Pan-Coronavirus Vaccine as well as toxin prediction for each epitope.

Epitope	IEDB ID	sequence	Protein	RF Score (95% CI)	MHC restriction	Assay description	Toxin
CTL1	1309147	YLQPRTFLL	Spike glycoprotein	0.66 (0.63:0.69)	H2 class I;HLA class I;HLA-A*02:01;HLA-A*24:02;HLA-B*07:02;HLA-B*08:01	IFNg release/TNFa release/qualitative binding/granzyme A release/granzyme B release/perforin release/cytotoxicity/activation/qualitative binding/proliferation/degranulation/dissociation constant KD/CCL4/MIP-1b release/IL-2 release	Non-toxin
CTL2	37473	LLLDRLNQL	Nucleoprotein	0.43 (0.39:0.47)	HLA class I/HLA-A*02:01/HLA-A2/Mamu-A1*026:01	Activation/cytotoxicity/IFNg release/pathogen burden after challenge/proliferation/qualitative binding/TNF release/TNFa release	Non-toxin
CTL3	60242	SPRWYFYYL	Nucleoprotein	0.75 (0.69:0.80)	H2-b class I;HLA class I;HLA class II;HLA-A*02:01;HLA-B*07:02;HLA-B*08:01;HLA-B35;HLA-B7	Activation/CCL4/MIP-1b release/cytotoxicity/degranulation/IFNg release/IL-2 release/pathogen burden after challenge/qualitative binding/TNF release/TNFa release	Non-toxin
CTL4	1310756	QYIKWPWYI	Spike glycoprotein	0.47 (0.41:0.54)	HLA class I;HLA-A*23:01;HLA-A*24:02;HLA-A24;HLA-C*06:02	Activation/cytotoxicity/degranulation/IFNg release/IL-2 release/qualitative binding/TNFa release	Non-toxin
CTL5	1309115	FTSDYYQLY	ORF3a protein	0.63 (0.55:0.70)	HLA class I;HLA-A*01:01;HLA-A*24:02	Activation/cytotoxicity/degranulation/IFNg release/qualitative binding	Non-toxin
CTL6	33667	KTFPPTEPK	Nucleoprotein	0.40 (0.34:0.46)	HLA class I;HLA-A*03:01;HLA-A*11:01;HLA-A*30:01;HLA-A*68:01	Activation/cytotoxicity/IFNg release/IL-2 release/qualitative binding/TNFa release	Non-toxin
CTL7	72048	VYIGDPAQL	Replicase polyprotein 1ab	0.26 (0.19:0.34)	HLA class I;HLA-A*02:01;HLA-A*24:02;HLA-A24;HLA-C*07:01	Activation/cytotoxicity/IFNg release/qualitative binding/TNFa release	Non-toxin
CTL8	190494	MEVTPSGTWL	Nucleoprotein	0.50 (0.41:0.59)	HLA class I;HLA-B*40:01;HLA-B*40:10;HLA-B40	Activation/cytotoxicity/degranulation/granzyme B release/IFNg release/perforin release/qualitative binding/TNF release/TNFa release	Non-toxin
CTL9	1310623	LTDEMIAQY	Spike glycoprotein	0.42 (0.35:0.50)	HLA class I;HLA-A*01:01;HLA-A*29:02;HLA-B*35:01;HLA-C*07:02	Activation/degranulation/granzyme B release/IFNg release/qualitative binding TNF release/TNFa release	Non-toxin
CTL10	1311180	LLYDANYFL	ORF3a protein	0.54 (0.48:0.60)	HLA class I;HLA-A*02:01	Activation/cytotoxicity/degranulation/IFNg release/qualitative binding/TNFa release	Non-toxin

**CTL11**	**1313269**	**NYNYLYRLF**	**Spike glycoprotein**	**0.65 (0.56:0.74)**	**H2-d class I;HLA-A*24:02**	**Activation/degranulation/IFNg release/IL-2 release/qualitative binding/TNFa release**	**Non-toxin**
**CTL12**	**16156**	**FIAGLIAIV**	**Spike glycoprotein**	**0.35 (0.31:0.40)**	**H2 class I;HLA class I;HLA-A*02:01;HLA-A2**	**Activation/cytotoxicity/degranulation/IFNg release/qualitative binding/T cell binding/TNFa release**	**Non-toxin**
**CTL13**	**1310934**	**VYFLQSINF**	**ORF3a protein**	**0.32 (0.24:0.42)**	**HLA-A*02:01;HLA-A*24:02;HLA-A24**	**Activation/cytotoxicity/IFNg release/qualitative binding**	**Non-toxin**
**CTL14**	**1311144**	**DTDFVNEFY**	**Replicase polyprotein 1ab**	**0.34 (0.25:0.44)**	**HLA class I;HLA-A*01:01**	**Cytotoxicity/IFNg release/qualitative binding**	**Non-toxin**
**CTL15**	**54725**	**RLQSLQTYV**	**Spike glycoprotein**	**0.35 (0.29:0.41)**	**HLA class I;HLA-A*02:01;HLA-A*02:03;HLA-B*13:02**	**Activation/degranulation/dissociation constant KD/IFNg release/qualitative binding/TNFa release**	**Non-toxin**
**CTL16**	**71663**	**VVFLHVTYV**	**Spike glycoprotein**	**0.15 (0.11:0.20)**	**HLA class I;HLA-A*02:01;HLA-A*02:06;HLA-C*16:02**	**Activation/IFNg release/qualitative binding**	**Non-toxin**
**CTL17**	**1074846**	**ALSKGVHFV**	**ORF3a protein**	**0.35 (0.28:0.42)**	**HLA class I;HLA-A*02:01**	**Activation/cytotoxicity/IFNg release/qualitative binding/T cell binding**	**Non-toxin**
**CTL18**	**1310872**	**TTDPSFLGRY**	**Replicase polyprotein 1ab**	**0.67 (0.59:0.73)**	**H2-b class I;HLA class I;HLA-A*01:01;HLA-A*03:01**	**Activation/cytotoxicity/degranulation/granzyme B release/IFNg release/pathogen burden after challenge/qualitative binding/TNF release/TNFa release**	**Non-toxin**

Table S11. The candidate immunodominant CTL-cell epitopes exhibited a high degree of conservation among SARS-CoV-2 VOCs and VOIs.

Epitopes	Sequence	conservancy
CTL6(WT)	KTFPPTEPK	100%
CTL6(variant1)	KTFPPIGPK	77.78%
CTL8(WT)	MEVTPSGTWL	100%
CTL8(variant1)	MEVTPSGTWF	90.00%
CTL8(variant2)	MEVTPSGTLL	90.00%
CTL10(WT)	LLYDANYFL	100%
CTL10(variant1)	LFYDANYFL	88.89%
CTL11(WT)	NYNYLYRLF	100%
CTL11(variant1)	NYNYQYRLF	88.89%
CTL11(variant2)	NNNYLYRLF	88.89%
CTL11(variant3)	NYNYRYRLF	88.89%
CTL11(variant4)	NYNYLYRLL	88.89%
CTL12(WT)	FIAGLIAIV	100%
CTL12(variant1)	FTAGLIAIV	88.89%
CTL13(WT)	VYFLQSINF	100%
CTL13(variant1)	VYFLQSMNF	88.89%

Table S12. The candidate immunodominant CTL-cell epitopes exhibited a high degree of conservation among zoonotic coronaviruses.

Epitopes	Sequence	conservancy	ID
CTL1(WT)	YLQPRTFLL	100%	QHD43416, MN996532,
CTL1(variant1)	YLAPRTFML	77.78%	MT121216
CTL1(variant2)	KLQPLTFLL	77.78%	KF917527, NC_019843
CTL1(variant3)	YLQQRTFLL	88.89%	MT040333, MT040335, MT040336, MT072864
CTL2(WT)	LLLDRLNQL	100%	AAP13445, AY572034, FJ211859, FJ588686, KF367457, KP886808,KT444582, MN996532, MT121216,
CTL2(variant1)	LLLDRLNAL	88.89%	MT040333, MT040334, MT040335, MT040336, MT072864
CTL3(WT)	SPRWYFYYL	100%	AAP13445, AY572034, FJ211859, FJ588686, KF367457, KP886808,KT444582, MN996532, MT040333, MT040334, MT040335, MT040336, MT072864, MT121216
CTL3(variant1)	LPRWYFYYL	88.89%	AAX76525, AIV41880, FJ647226, KM349742, KT368891, MN514967

CTL3(variant2)	DPRWYFYYL	88.89%	KF636752
CTL3(variant3)	APRWFFYYT	66.67%	EF065505
CTL3(variant4)	APRWYFYYT	77.78%	KF917527, MH002342, NC_019843
CTL3(variant5)	NARWYFYYT	66.67%	MG762674
CTL3(variant6)	NPRWYFYYT	77.78%	MT350598
CTL4(WT)	QYIKWPWYI	100%	QHD43416, KF636752, MN996532, MT040333, MT040334, MT040335, MT040336, MT072864, MT121216,
CTL4(variant1)	QYIKWPWYV	88.89%	AAP13441, AY572034, FJ211859, FJ588686, KF367457, KP886808, KT444582
CTL4(variant2)	YYVKWPWYV	66.67%	AIV41879, KT368891, MN514967
CTL4(variant3)	NYIKWPWWV	66.67%	YP_003767
CTL4(variant4)	YYNKWPWYV	66.67%	EF065505
CTL4(variant5)	MYVKWPWYV	66.67%	FJ647226, KM349742
CTL4(variant6)	TYIKWPWWV	66.67%	KY983587
CTL4(variant7)	QTIKWPWYV	77.78%	MG762674
CTL5(WT)	FTSDYYQLY	100%	QHD43417, MT121216
CTL5(variant1)	FTSECYQLY	77.78%	MT040333, MT040334, MT040335,MT040336, MT072864
CTL6(WT)	KTFPPTEPK	100%	QHD43423, AY572034, FJ211859, FJ588686, KF367457, KP886808,KT444582, MN996532, MT040333, MT040334, MT040335, MT040336, MT072864, MT121216,
CTL6(variant1)	KNFPPPEPK	77.78%	MG762674
CTL7(WT)	VYIGDPAQL	100%	AAP13442, AAX76519, AIV41876,QHD43415, AY572034, FJ588686, KF367457, KM349742, KP886808, KT368891, KT444582, MN514967, MN996532, MT040333, MT040334, MT040335, MT040336, MT072864, MT121216

CTL7(variant1)	VYVGDPQQL	77.78%	YP_003766, KY983587, NC_028752
CTL7(variant2)	VYVGDPAQL	88.89%	EF065505, KF636752, KF917527, MG762674, MH002342, MT350598, NC_019843
CTL8(WT)	MEVTPSGTWL	100%	AAP13445, AY572034, FJ211859, FJ588686, KF367457, KP886808,KT444582, MN996532, MT040333, MT040334, MT040335, MT040336, MT072864, MT121216,
CTL9(WT)	LTDEMIAQY	100%	QHD43416, MN996532, MT040333, MT040334, MT040335, MT040336, MT072864, MT121216,
CTL9(variant1)	LTDDMIAAY	77.78%	AAP13441, AY572034, KF367457, KP886808, KT444582
CTL9(variant2)	LTDEMVAAY	77.78%	FJ211859
CTL9(variant3)	LTDEMIAAY	88.89%	FJ588686
CTL10(WT)	LLYDANYFL	100%	QHD43417, MT040333, MT040334, MT040335, MT040336, MT072864, MT121216
CTL10(variant1)	LLYEANYFV	77.78%	AY572034
CTL10(variant2)	LLYDANYFV	88.89%	FJ211859, KP886808, KT444582
CTL11(WT)	NYNYLYRLF	100%	QHD43416, MT121216,
CTL11(variant1)	NFNYLYRLF	88.89%	MN996532
CTL11(variant2)	NYGYLYRLF	88.89%	MT040333, MT040335, MT040336, MT072864
CTL12(WT)	FIAGLIAIV	100%	AAP13441, QHD43416, AY572034, FJ211859, FJ588686, KF367457, KF636752, KT444582
CTL12(variant1)	FIAGLIAII	88.89%	MN996532, MT040333, MT040334, MT040335, MT040336, MT072864, MT121216
CTL13(WT)	VYFLQSINF	100%	QHD43416, MT121216
CTL13(variant1)	VYFLQSVNA	77.78%	MT040333, MT040334, MT040335, MT040336, MT072864
CTL14(WT)	DTDFVNEFY	100%	QHD43415, MN996532, MT040333, MT040334, MT040335, MT040336, MT072864, MT121216

CTL14(variant1)	DPAFVNEFY	77.78%	KM349742
CTL15(WT)	RLQSLQTYV	100%	AAP13441, QHD43416, AY572034, FJ211859, FJ588686, KF367457, KP886808, KT444582, MN996532, MT040333, MT040334, MT040335, MT040336, MT072864, MT121216
CTL15(variant1)	RLQVLQTFV	77.78%	KF636752
CTL16(WT)	VVFLHVTYV	100%	AAP13441, QHD43416, AY572034, FJ211859, FJ588686, KF367457, KP886808, KT444582, MN996532, MT040333, MT040334, MT040335, MT040336, MT072864, MT121216
CTL17(WT)	ALSKGVHFV	100%	QHD43417,MT121216
CTL17(variant)	ALSKGVHFA		MT040333, MT040334, MT040335, MT040336, MT072864
CTL18(WT)	TTDPSFLGRY	100%	QHD43415, MN996532
CTL18(variant1)	TLDESFLGRY	80.00%	AAP13442, AY572034, FJ588686, KF367457, KP886808, KT444582
CTL18(variant2)	TIDESFLGRY	80.00%	FJ211859
CTL18(variant3)	TTDESFLGRY	90.00%	MT040333, MT040334, MT040335, MT040336, MT072864
CTL18(variant4)	TADAAFLHRY	60.00%	MH002342
CTL18(variant5)	TTDASFLGRY	90.00%	MT121216
